# Developmental effects of sulfated thyroid hormones in sea urchin skeletogenesis suggest activation of non-canonical thyroid hormone signaling pathway

**DOI:** 10.3389/fendo.2025.1648899

**Published:** 2025-08-21

**Authors:** Katherine Tieman, Andreas Heyland

**Affiliations:** Integrative Biology, University of Guelph, Guelph, ON, Canada

**Keywords:** non-genomic, integrin receptor, skeletogenesis, sea urchin embryo, AlphaFold, binding affinity, thyroid hormone metabolites

## Abstract

Thyroid hormones (THs) are essential regulators of metabolism, homeostasis, and development in metazoans. The canonical genomic pathway involves THs binding to nuclear thyroid hormone receptors (NTHRs), which modulate gene expression in vertebrates. In contrast, non-genomic pathways involve THs interacting with membrane-bound or cytoplasmic receptors. One such pathway includes TH binding to the RGD-binding integrin dimer αVβ3, which activates the Mitogen-Activated Protein Kinase (MAPK) cascade, influencing cancer cell proliferation, metastasis, and angiogenesis. Both T4 and sulfated thyroid hormones (STHs) have been identified as actual and putative ligands in this pathway respectively. In the sea urchin *Strongylocentrotus purpuratus*, T4 and to a lesser extent T3 accelerate biomineralization—the formation of skeletal structures during embryonic and larval development—by modulating the activity of key transcription factors involved in this process. RGD peptides, potential ligands for the sea urchin integrin αPβG, can inhibit T4-induced effects, suggesting a role for integrin-mediated MAPK signaling (ERK1/2). This study examines whether STHs have developmental roles in sea urchin embryonic skeletogenesis and whether they bind to the αPβG integrin dimer *in silico*, a TH receptor candidate in sea urchins. Our findings show that STHs, like T4, accelerate the onset of skeletogenesis and increase the frequency of ectopic spicule formation, particularly near ectodermal cells. Homology modeling indicates that the αPβG integrin binds both T4 and STHs with high affinity, whereas no strong binding was observed between TH metabolites and the NTHR in sea urchins. We conclude that STHs have a developmental function in sea urchin skeletogenesis, likely mediated by the αPβG integrin rather than the NTHR. This represents the first documented developmental role of STHs and highlights the importance of non-canonical TH signaling in invertebrate development, encouraging further exploration of TH pathways in non-chordate animals.

## 1 Introduction

Thyroid hormones (THs) are signaling molecules that play essential roles in regulating metabolism, homeostasis, and development across vertebrates and several invertebrate lineages. Structurally, THs are iodinated derivatives of di-tyrosine, with over 26 known metabolites, including thyroxine (T4), triiodothyronine (T3), and various sulfated forms (STHs), including ST4 (sulfated thyroxine) and ST3 (sulfated 3,5,3′-Triiodothyronine) (1). While their endocrine functions are well-characterized in mammals and other vertebrates, emerging evidence suggests that THs also function as signaling molecules in a range of invertebrates (2–5).

The evolutionary origins of TH signaling likely predate the emergence of metazoans. It has been hypothesized that early animals may have utilized exogenous THs from dietary sources as vitamins, which could have facilitated the evolution of endogenous TH signaling pathways (3, 4). In animals, two primary mechanisms of TH action have been identified: The genomic pathway involves the binding of T3 to nuclear thyroid hormone receptors (NTHRs), which heterodimerize with retinoid X receptors (RXRs) to regulate gene transcription involved in development and metabolism (6, 7). In contrast, the non-genomic pathway operates through membrane-associated receptors, notably the integrin αVβ3 in humans (8). This integrin dimer is part of a sub-group of extracellular matrix receptors composed of specific α and β subunits that recognize and bind the RGD peptide—a tripeptide motif known for its diverse signaling roles, particularly in mediating cell adhesion (9). αVβ3 mediates rapid cellular responses by activating the MAPK/ERK1/2 signaling cascade upon TH binding in mammalian angiogenesis and has also been shown to play a key role in cancer metastasis (10, 11). STHs, iodinated di-tyrosines, which are formed by sulfotransferases via sulfation, have been shown to bind with higher affinity to this integrin receptor than their non-sulfated counterparts *in silico* (12). These findings are noteworthy as STHs have generally been viewed as inactive metabolites of T4 and T3. Intriguingly, the signaling of THs via integrin receptors is not only conserved across metazoans but is also susceptible to disruption by environmental contaminants such as organophosphate esters, which mimic THs and induce neurodevelopmental toxicity via integrin-mediated ERK1/2 activation in zebrafish (13, 14).

In invertebrates, THs have been implicated in diverse physiological and developmental processes, including morphological remodeling, tissue reorganization, and biomineralization, particularly in mollusks, echinoderms, tunicates, and cephalochordates (4, 5, 15). In sea urchins, iodine uptake occurs via hydrogen peroxide-facilitated diffusion, and thyroxine has been localized to endodermal neurons during embryogenesis (16, 17). Single-cell transcriptomic analyses have identified the expression of genes involved in TH synthesis and signaling, including thyroid-stimulating hormone (TSH), which is expressed in the foregut and skeletal cells during gastrulation, although its function appears to be independent of canonical TH signaling (18).

Sea urchin skeletogenesis, a well-characterized developmental process, provides a valuable model for studying TH signaling. During gastrulation, primary mesenchyme cells (PMCs) migrate and form a ring around the vegetal plate, initiating the formation of calcite spicules through a VEGF-regulated gene regulatory network (19–21). THs, particularly T4, have been shown to accelerate spicule formation via ERK1/2 phosphorylation, implicating non-genomic signaling through integrin receptors (11, 17, 22). This effect is partially inhibited by RGD peptides, which compete for integrin binding, further supporting the role of integrin-mediated TH signaling in sea urchin development.

Fluorescently labeled T4 binds to PMC membranes, and both, NTHRs and the integrin subunit αP are expressed in these cells during gastrulation (11, 18, 22). However, transcriptomic data suggests that TH-induced skeletogenic effects in the embryo are largely independent of nuclear receptor signaling (11). To further elucidate the mechanisms of TH action, we investigated the effects of STHs on sea urchin embryogenesis and specifically the initiation and elongation of the larval skeleton. Our results demonstrate that STHs exert stronger effects on skeletogenesis than non-sulfated forms. *In silico* modeling revealed that the sea urchin integrin αPβG binds ST4 and T4 with affinities comparable to the human αVβ3 integrin, reinforcing the similarity between integrin mediated TH signaling between mammals and sea urchins.

## 2 Materials and methods

### 2.1 Animal care and use

Adult *S. purpuratus* individuals were collected via diving by Monterey Abalone Company (Monterey, CA) and shipped to the Hagen Aqualab at the University of Guelph, ON. They were housed in tanks in a recirculating artificial seawater system where conditions simulated perpetual winter to maintain spawning competency with temperature maintained at 12-14°C, salinity at 32–34 ppt and a 16(D):8(L) hour light cycle. *S. purpuratus* adults were fed *Kombu* spp. kelp three times a week.

Pre-fertilization, we filtered and UV-treated artificial seawater made with Instant Ocean and added 0.02-0.03 g/L pH 8.3 Reef Buffer and cooled it to 14°C over 24 hours, which is referred to as FASW. To obtain gametes, adult spawning was induced via a combination of injecting 0.5–1 mL of [0.5 M] KCl salt (Fisher Scientific P330) mixed in milliQ water and shaking. A pipette was used to collect sperm from males and then diluted at 1–2 drops of sperm per 10 mL FASW to a concentration conducive to avoiding polyspermy during titration. Eggs were collected by inverting the female over a beaker of FASW and then rinsed twice with FASW to remove abnormally developed eggs and debris. The diluted sperm was titrated into the beaker of eggs until either 90% of eggs developed a fertilization envelope or three subsequent additions of sperm do not yield a higher fertilization success rate to avoid polyspermy. Eggs were then transferred to a 1L beaker of FASW and held at 14°C until they hatched. At hatch, embryos were transferred to a new container to remove unhatched eggs and used immediately in experiments or cultured.

### 2.2 Thyroid hormone stock preparation

T4, T3, rT3 were obtained from Sigma-Aldrich (product numbers T2376, T2877, and T0281), ST4 from MCE (cat. number HY-101406), and ST3 from LGC (CAS number 31135-55-4) and stored according to manufacturer directions. THs were dissolved in 100% DMSO to create stock solutions at 10 mM and stored at -20°C and protected from light until they were used. DMSO and biologically inactive rT3 function as negative controls. An aliquot of 100% DMSO was stored at -20°C and used in the control treatment. Working stocks of the hormones and DMSO were created before each experiment via dilution with FASW and were used immediately.

### 2.3 Pharmacological assay of STH exposure measuring effects on skeletogenesis in embryos

**Figure 1:**
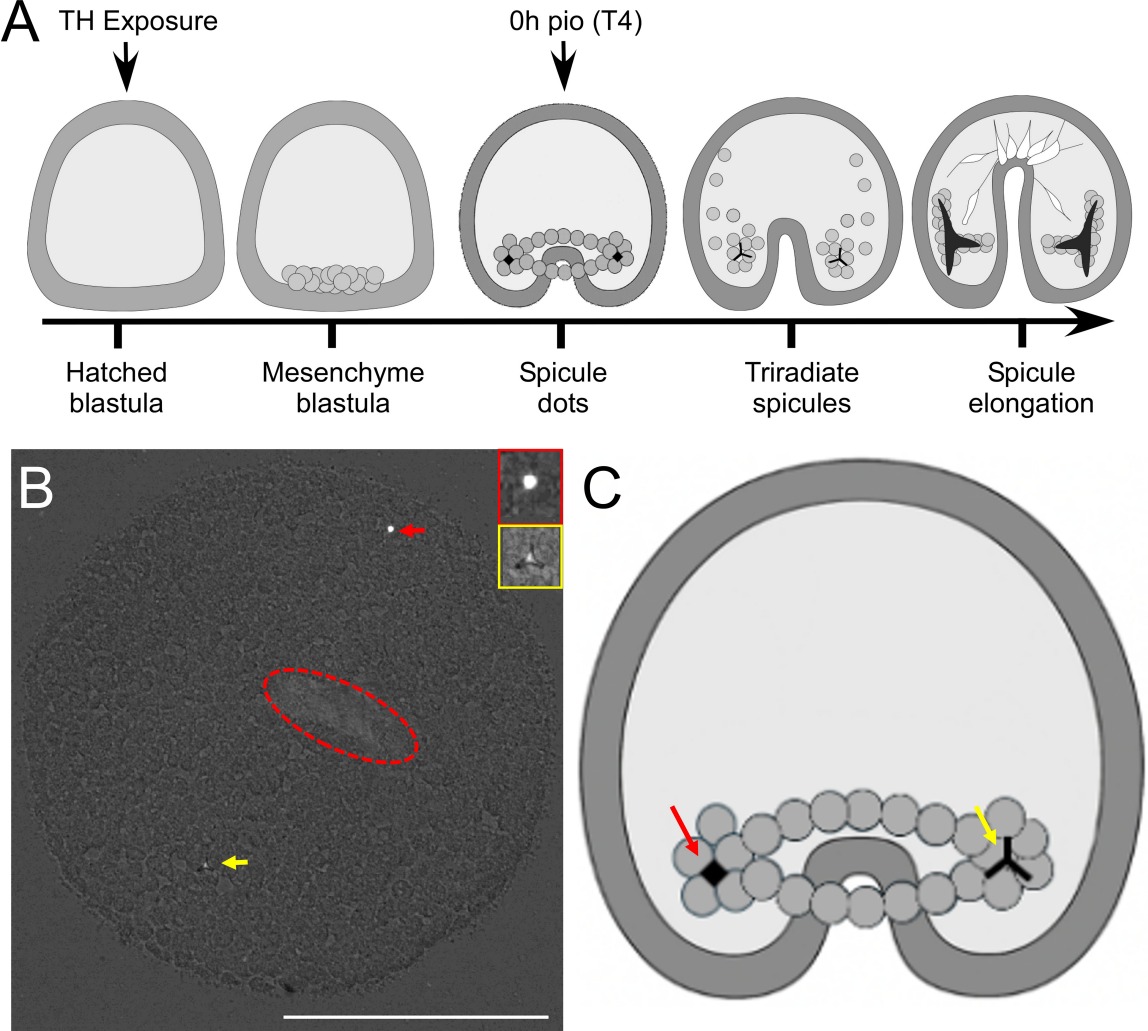
Experimental design and assessment of spicule formation, using polarized light microscopy. **(A)** Embryos were exposed to THs and monitored until 50% of embryos had developed spicules in the thyroxine (T4) treatment. We identify this time point as 0h pio (post initial observation). To capture spicule dot formation (early spicule), we removed embryos from cultures and imaged them under polarized light, after flattening them. Early spicules [**(B)** – red arrow] and tri-radiate spicules [**(B)** – yellow arrow] were visible in these preparations. The red ellipsoid marks the location of the archenteron. **(C)** shows sketch of these types of skeletal structures in the embryo at the spicule do and triradiate spicule stage. White scale bar represents 100µm.

Post-fertilized embryos were left to develop for 18 hours and then monitored for signs of hatching. Upon hatching, embryos were poured into a new container, and the concentration of hatched embryos was assessed. Embryos were mixed and randomly separated into falcon tubes at 20 embryos/mL and exposed to the corresponding treatment (N = 960). Embryos at the gastrula stage do not yet have a functional gut and were thus not fed before or during the experiment. The experimental protocol used for these exposures is outlined in **Figure 1A**. Treatments included T4, T3, ST4, ST3, and rT3 and DMSO controls at [10–^7^ M] and [10–^9^ M] and all combinations of treatment and concentration were measured at four timepoints. These concentrations were used based on extensive previous experiments, including dose responses on skeletogenesis of sea urchins with T4, T3, T2, TRIAC and other iodinated tyrosines (2, 3, 10, 11, 16, 17, 22–28), indicating that these concentration result in physiological responses. Our own work on assessing binding affinities of T4 and other THs to the putative membrane receptor. Suggest a Kd of T4 of around 10^-8^M in *S. purpuratus*. Immediately following exposure, embryos were transferred to 24-well plates with 1 mL/well (n = 20) and remained in an incubator at 14°C for the duration of the experiment. Each treatment was replicated in four wells at each timepoint with a nested design so that well effect along with individual effect could be assessed. To determine the initial timepoint, additional embryos exposed to 10–^7^ M T4 were monitored until ~50% developed spicules (early skeleton). T4 was chosen as it has previously been shown to accelerate skeleton formation greater than T3 during *S. purpuratus* gastrulation (11, 22). At ~50% T4 early spicule formation, embryos from four wells per treatment were fixed with 500 μL ethanol/well and briefly observed to ensure 100% mortality [10.9 hours post exposure (hpe) = 0 hours post initial observation (hours pio)]. Embryos from the remaining wells continued to develop and were fixed in ethanol at 4-hour increments with 4 wells/treatment at 14.9 hpe (4 hours pio), 18.9 hpe (8 hours pio), and 22.9 hpe (12 hours pio). Fixed embryos were stored in the fridge and imaged within two days of mortality to avoid spicule degradation. Embryos were transferred to poly-L-lysine coated slides, and excess media was removed to ensure that when the coverslip was placed, the embryos remained under the cover slide and were compressed, allowing for clearer visualization of the spicule structures. Slides were imaged on a Nikon Ti2 Eclipse microscope using polarized light which increases the contrast between the crystalized structure of spicules and cells. Despite being compressed, Z-stack images of each embryo were taken so that no early spicules were missed (**Figure 1B**). Images were used to record the number of early-spicules (defined as small roughly spherical spicules, **Figure 1B**) and tri-radiate spicules (defined as a spicule that had branches extending in three directions from a center point, **Figure 1B**), and spicule length was measured via ImageJ for each tri-radiate spicule as the sum of the three branches (**Figures 1B, C**). The staging scheme for skeletal stage included a value of one assigned for any individuals with no spicules, a value of two assigned to individuals with any number of early spicules with no spicule extension, and a value of three assigned for individuals with any number of tri-radiate spicules. Embryos were considered to have ectopic spicules if more than two spicules of any type were observed. Ectopic spicules were further analyzed based on potential spicule location. Based on positioning and length, two spicules were designated primary spicules. Additional spicules were deemed ectopic and were classified as associated with the position of one of the primary spicules, possibly associated with a primary spicule, if they were close to the primary spicule but not at the base of spicule, directly in between both primary spicules, or if there was evidence to suggest that it was located along the syncytial ring of PMCs, or not associated with the primary spicules.

### 2.4 Identification of orthologous receptors in non-human species

BLAST searches using the human receptor sequences were performed on the UniProt database (29) to identify orthologs to the human receptors from selected species, to verify gene annotations, and obtain sequence similarity percentages. To further confirm that the identified receptors are true orthologs, we used the OMA browser (https://omabrowser.org/oma/home/) to cross-check receptors within the Hierarchical Orthologous Groups (HOG) category, which clusters genes based on inferred orthologous relationships (30). This verification step provided additional confidence in the evolutionary relationships of the receptors used in further modeling analyses. For *H. sapiens* αvβ3 we used proteins P06756 (αv) and P05106 (β3). For sea urchin αPβG we used proteins Q9U6S1 (αP) and P92163 (βG). FASTA sequences for the following receptors were obtained from UniProt (The UniProt Consortium, 2023) based on the results of the BLAST and ortholog searches: *S. purpuratus* THRβ (A0A7M7N2J7), *S. purpuratus* RXR (A0A7M7PMF3), *S. purpuratus* αP subunit (Q9U6S1), *S. purpuratus* βG subunit (P92163), *H. sapiens* THRβ (P10828), *H. sapiens* RXRα (P19793), *H. sapiens* αv subunit (P06756), *H. sapiens* subunit β3 (P05106), *B. floridae* THR (A7L5U9), *B. floridae* RXR (Q8MX78), *C. intestinalis* nuclear receptor 1 (H2XS16), *C. intestinalis* RXR (Q4H2U9), *C. gigas* THR (A0A0F6V015), *C. gigas* RXR (K1PXX3), *D. rerio* THRβ (Q9PVE4), and *D. rerio* RXRαβ (F1Q4V9).

### 2.5 Protein model generation

For integrin receptor models, the ligand binding site lies between the two subunits and therefore does not bind specifically to either monomer (8, 31). Therefore, the dimeric models were constructed for these models using the web-based Alphafold2/ColabFold structure prediction tool (tamarind.bio) using the multimer setting (32). Mn^2+^ ion binding sites for integrin dimers αvβ3 and αPβG were predicted using default parameters (33).

For NTHRs, the THR or homologous sequence from each species was submitted to a web-based Alphafold2/ColabFold structure prediction tool (tamarind.bio) using the monomer setting (32). This webserver was used over ColabFold (the publicly accessible version of AlphaFold2 on Google Colab) because it has a higher amino acid sequence length capacity for multimer predictions. This approach still maintains but minimizes potential biases introduced in classical homology modeling as it creates a custom MSA instead of basing the model off a single structure, and uses AI detected patterns in protein folding to predict the structure. However, it remains biased towards proteins with crystal structures, of which many are vertebrates which may impact predicted results for invertebrate sequences with no closely related crystal structures available (34). The AlphaFold2-based tool generated five models of the predicted structure for each receptor based upon structures with related sequences, and the top-ranking model based on two internal scoring systems were used for all downstream modeling work (32). The THR sequences were also submitted under the multimer setting in the Alphafold2/ColabFold structure prediction tool with the RXR sequence of the corresponding species to compare monomer versus dimer docking scores, as dimerization with RXR is known to enhance binding at the THR site (32).

Models and crystal structures were visualized using PyMOL, and per-residue pLDDT scores were generated for each model to assess model quality (35). For structure validation, crystal structures for the human receptors were obtained through the RCSB (RCSB.org (36); protein data bank (THRβ monomers: 3gws (37), THRβ dimers: 4zo1 (38); αvβ3 dimers: 1l5g (39). We then used PyMOL’s align feature to overlay the models onto the crystal structures and visually inspected the structures for any poorly predicted regions that might conflict with or block binding.

### 2.6 Evaluating structural conservation of TH receptors across taxa

We generated multiple sequence alignments with ClustalW (40) with default settings for each subunit type for the integrins to compare amino acid residues between these species.

For the NTHRs, we generated a multiple sequence alignment with ClustalW (40) with default settings. The resulting multiple sequence alignment was then uploaded to the Consurf server (41), also using its default parameters, to assess residue conservation of the select species. Consurf produced PyMOL coloring scripts that grouped residues into distinct conservation bins and allowed visualization of conservation patterns directly on the human protein structure.

### 2.7 Protein ligand docking and prediction of binding affinity for integrins

For integrin docking predictions, CBDock2 could not be used as it does not accept models that include ions, which are theoretically crucial to TH binding to integrin (34). We instead used HADDOCK webserver (42) to predict ligand position. Of available docking software, we chose HADDOCK primarily due to the ability to accept ions within the submitted structure, a rare feature of docking software, along with its public accessibility. To predict binding position, receptor and ligand structures were uploaded to the HADDOCK webserver. According to the small molecule binding site screening protocol (https://www.bonvinlab.org/education/HADDOCK24/HADDOCK24-binding-sites/), a few settings were changed from default, which included turning on random patches define randomly ambiguous interaction restraints from accessible residues, setting the number of structures for rigid body docking to 10000, setting the number of structures for semi-flexible refinement to 400, setting the number of structures for the final refinement to 400, and setting the RMSD cutoff for clustering to 2.0. However, HADDOCK does not directly calculate predicted binding affinity. For this, the best scoring predicted ligand position based on internal scoring from HADDOCK were uploaded to PRODIGY-Ligand using default parameters (43).

### 2.8 Protein ligand docking and prediction of binding affinity for NTHRs

Ligand structures were obtained from (12) and used for all docking models and calculations. For the NTHRs, we investigated ligand positioning and binding affinity within the buried ligand-binding pocket using CBDock2 (44). CBDock2 was used to conduct both blind docking and template-based docking to maximize the potential to find the best ligand position. Many types of software used for protein-ligand docking cannot detect binding pockets buried within the protein, a limitation not found in CBDock2, making it an ideal choice for this receptor, based in our own experience and work with the software. Of available docking software that can detect buried binding pockets, we chose CBDock2 due to its high performance against other similar software, its dual approach to docking using blind and template-based docking, it’s frequent use in recent literature, with over 600 citations to date, and its public accessibility (44). Assessment of CBDock2 ligand binding quality showed that it generates binding poses within a small range of error from x-ray crystal structure binding poses and outcompetes other competitors (44, 45). We increased the number of cavities for blind docking to 20 results to gain results for non-human species where binding pockets were not as conserved, thus requiring more generated binding pockets to identify the location of the binding pocket known from humans. The template database in CBDock2 only contained two of the *H. sapiens* crystal structures available [1y0x - (46), 3gws - (47)], and therefore, we submitted two other templates, 3gws - (37) and 4zo1 - (38), through the template user submission tool on CBDock2 to assess binding affinity for all potential known TH binding poses. The best binding affinity scores based on all poses generated for the binding pocket of interest were reported.

### 2.9 Statistical Analysis

Dependent variables for the embryonic pharmacological assay included total number of spicules, number of early spicules (spicule dots), number of tri-radiate spicules, number of ectopic spicules, presence of ectopic spicules, total length of spicules, stage of development, and ectopic spicule location. Total number of spicules, number of early spicules, and stage of development assess whether STHs accelerated spicule formation, particularly at the 0 and 4 hours pio. Number of tri-radiate spicules, and total length of spicules, particularly at 8 and 12 hours pio, assess whether STHs accelerate spicule elongation. The number of ectopic spicules and presence of ectopic spicules assess whether STHs increase ectopic spicule formation.

All statistical tests were conducted using SPSS 25. In all cases, a p < 0.05 was considered significant. For all variables, we assessed the distribution of the data through P-P, Q-Q plots, and histograms. Except for spicule length, all variables were ordinal, and the data was not normally distributed. We assessed homogeneity of variance for spicule length using Levene’s test. We split the dataset by time and concentration as these were independent samples and conducted a Kruskal-Wallis test using all pairwise comparisons with Dunn’s tests with Bonferroni corrections for each variable. To assess differences in TH effects between the two concentrations, we split the dataset by time and treatment and then conducted Mann-Whitney U tests for each variable. In all cases, a p < 0.05 was considered significant.

## 3 Results

### 3.1 STHs accelerate embryonic skeletogenesis comparable to non-sulfated THs

**Figure 2:**
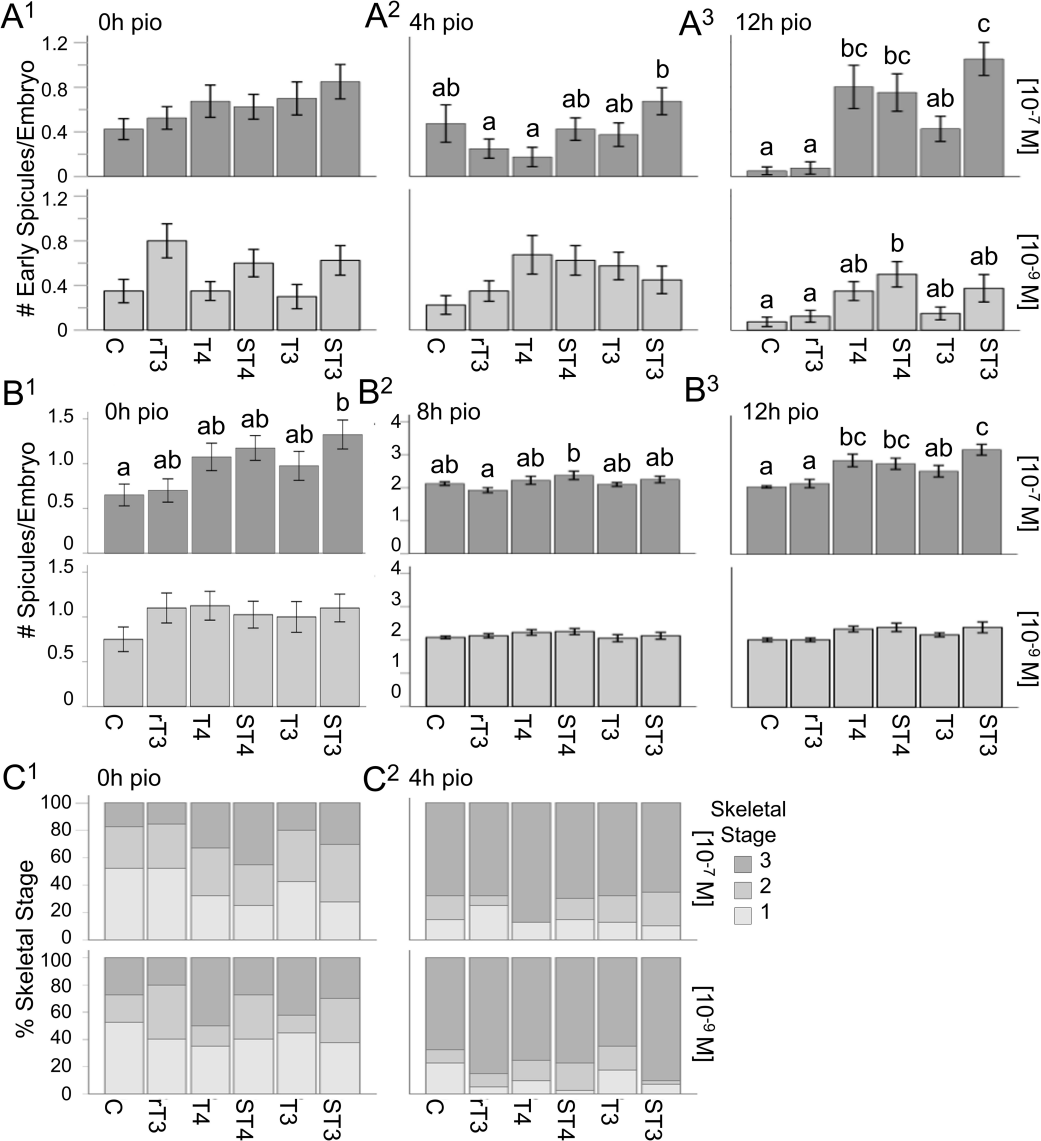
Sulfated thyroid hormones (STHs) accelerate spicule formation in sea urchin embryogenesis. **(A^1^–A^3^)** Number of early spicules for all treatments at 0, 4, and 12h pio, for both treatment concentrations. **(B^1^–B^3^)** Number of spicules for all treatments at 0, 8, and 12h pio, for both concentrations. **(C^1^, C^2^)** Skeletal stages for all treatments at 0 and 4h pio, for both treatment concentrations (1: no spicules; 2: any number of early spicules with no spicule extension; 3: any number of tri-radiate spicules). Error bars represent ± 1 unit se. Letters above bars represent significant differences between treatments (p<0.05). Statistical comparisons are summarized in **Supplementary Data Sheet 1**.

We tested TH effects on a range of skeletal parameters at four discrete time points starting from the initial point of observation (pio – see also **Figure 1A**) to assess the timing of initiation of skeleton formation (**Figure 2** - # of early spicules/embryo, # of spicules/embryo and skeletal stage). TH treatment at 10^-7^M resulted in a significant effect of treatment on the number of early spicules/embryo (**Figure 2A^1-3^**) when tested at 4h pio (H_5_ = 16.29, p = 0.006, η_2_ = 0.048) and 12h pio (H_5_ = 57.26, p < 0.001, η_2_ = 0.22); **Figure 2A^1^**. At the low concentration (10^-9^M) a significant effect of treatment was found at 0h pio (H_5_ = 12.76, p = 0.026, η_2_ = 0.033), 4h pio (H_5_ = 11.62, p = 0.04, η_2_ = 0.028); (**Figure 2A^1^**), and 12h pio (H_5_ = 18.19, p = 0.003, η_2_ = 0.056), (**Figure 2A^3^**). The results of the *post-hoc* comparisons between treatment groups for all time points are summarized in **Supplementary Data Sheet 1**, **Supplementary Table S1** and indicated with letters on the graphs (**Figure 2A^1-3^**). Specifically, we found that 10^-7^M ST3 at 4h pio resulted in more early spicules/embryo compared to rT3 and T4 (**Figure 2A^2^**). 10^-7^M T4, ST4 and ST3 resulted in more early spicules/embryo compared to the control and rT3 at 12h pio (**Figure 2A^3^**). At the lower concentration (10^-9^M) ST4 treatment resulted in more early spicules compared to the control and rT3 at 12h pio (**Figure 2A^3^**). The results of the dose-response analysis between concentrations for all time points are summarized in **Supplementary Data Sheet 1**, **Supplementary Table S2**, and indicated with asterisks on the graphs in **Supplementary Data Sheet 2**. Specifically, we found that at 0h pio a higher concentration of T3 resulted in more early spicules/embryo compared to the lower concentration. At 4h pio, a higher concentration of T4 resulted in fewer early spicules/embryo and at 12h pio, a higher concentration of ST3 resulted in more early spicules/embryo compared to the lower concentration.

When analyzing the total number of spicules/embryo (**Figure 2B^1-3^**) we found treatment effects for 0h pio (H_5_ = 15.49, p = 0.008, η_2_ = 0.045); (**Figure 2B^1^**), 8h (H_5_ = 12.65, p = 0.027, η_2_ = 0.033); (**Figure 2B^2^**), and 12h (H_5_ = 58.70, p < 0.001, η_2_ = 0.23); (**Figure 2B^3^**), for the high concentration (10^-7^M) but not for the low concentration (10^-9^M). The results of the *post-hoc* comparisons between treatment groups for all time points are summarized in **Supplementary Data Sheet 1**, **Supplementary Table S3**, and indicated with letters on the graphs (**Figure 2B^1-3^**). Specifically, we found that ST3 resulted in more spicules/embryo at 0h pio compared to the control (**Figure 2B^1^**). At 8h pio, ST4 treatment resulted in more spicules per embryo compared to rT3 (**Figure 2B^2^**). At 12h pio, T4, ST4 and ST3 all resulted in more spicules/embryo compared to the control and rT3, and ST3 also resulted in more spicules/embryo compared to T3 (**Figure 2B^3^**). The results of the dose-response analysis between concentrations for all time points are summarized in **Supplementary Data Sheet 1**, **Supplementary Table S2**, and indicated with asterisks on the graphs (**Supplementary Data Sheet 2**). Specifically, we found that at 8h pio, a higher concentration of rT3 resulted in more spicules/embryo compared to the lower concentration. We also found that at 12h pio, a higher concentration of T4 and ST3 resulted in more spicules/embryo compared to the lower concentration.

Our staging scheme of skeletogenesis includes the following stages: *Stage 1* represents embryos without any spicules, *stage 2* represents embryos with any number of early spicules but no spicule extension and *stage 3* represent embryos with any number of tri-radiate spicules. TH treatment at the higher concentration (10^-7^M) resulted in significant effects on skeletal stage when tested at 0h pio (H_5_ = 16.49, p = 0.006, η_2_ = 0.049); (**Figure 2C^1^**). At the low concentration (10^-9^M) a significant effect of treatment was found at 4h pio (H_5_ = 11.12, p = 0.049, η_2_ = 0.026); (**Figure 2C^1^**). The results of the *post-hoc* comparisons between treatment groups for all time points are summarized in **Supplementary Data Sheet 1**, **Supplementary Table S4**, and indicated with letters on the graphs (**Supplementary Data Sheet 2**). Specifically, we found that ST4 resulted in a higher average skeletal stage at 0h pio (10^-7^M) compared to the control and rT3 (**Figure 2C^1^**). The results of the dose-response analysis between concentrations for all time points are summarized in **Supplementary Data Sheet 1**, **Supplementary Table S2**, and indicated with asterisks on the graphs (**Supplementary Data Sheet 2**). Specifically, we found that at 4h pio, a higher concentration of rT3 resulted in a lower average skeletal stage compared to the lower concentration (**Figure 2C^1^**).

**Figure 3:**
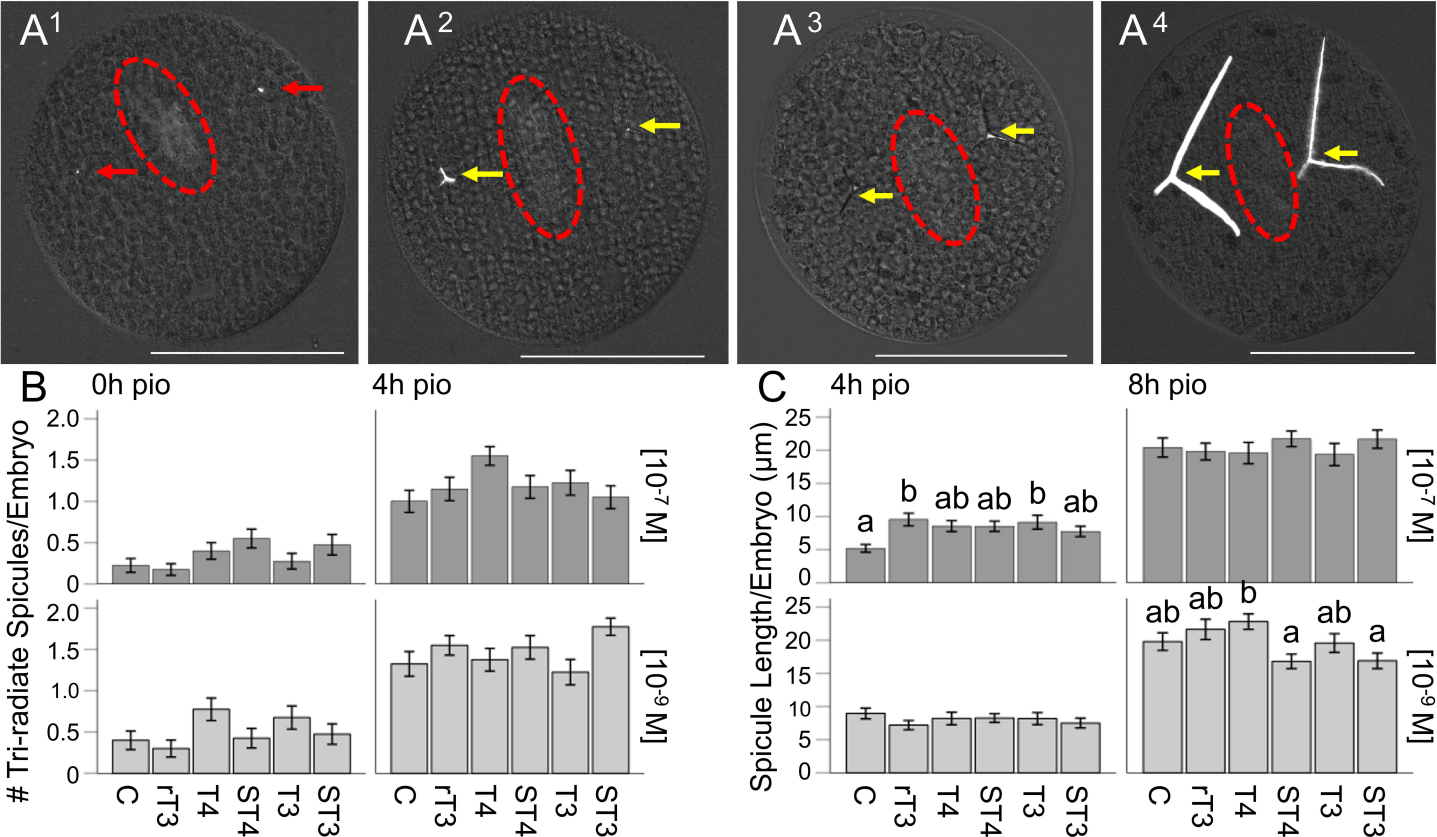
STH treatment does not result in increased skeletal elongation in sea urchin embryos. **(A^1^–A^4^)** Representative images of elongating tri-radiate spicules for 0h, 4h, 8h and 12h pio. **(B)** Number of tri-radiate spicules for all treatments at 0 and 4h pio, for both concentrations. **(C)** Spicule length for all treatments at 4 and 8h pio, for both concentrations. Red arrows indicate spicules, yellow arrows indicate tri-radiate spicules. The red ellipsoids mark the location of the archenteron. White scale bar represents 100µm, error bars represent ± 1 unit se. Letters above bars represent significant differences between treatments (p<0.05). Statistical comparisons are summarized in **Supplementary Data Sheet 1**.

We tested TH effects on the timing of elongation of skeletons (see **Figure 3A^1-4^** for representative images of skeleton elongation; **Figure 3B** - # of tri-radiate spicules/embryo) and the elongation of the skeleton (**Figure 3C** – spicule length) at four discrete time points starting from the initial point of observation (pio). TH treatment at the high concentration (10^-7^M) resulted in a significant effect of treatment on the number of tri-radiate spicules/embryo, when tested at 0h pio (H_5_ = 11.49, p = 0.042, η_2_ = 0.028); (**Figure 3B**). At the low concentration (10^-9^M) a significant effect of treatment was found at 0h pio (H_5_ = 11.13, p = 0.049, η_2_ = 0.026); (**Figure 3B**). The results of the *post-hoc* comparisons between treatment groups for all time points did not find any statistically significant differences for the number of triradiate spicules (**Supplementary Data Sheet 1**, **Supplementary Table S5**). The results of the dose-response analysis between concentrations for all time points are summarized in **Supplementary Data Sheet 1**, **Supplementary Table S6**, and indicated with asterisks on the graphs (**Supplementary Data Sheet 2**). Specifically, we found that at 0h pio a higher concentration of T4 and T3 resulted in fewer tri-radiate spicules/embryo compared to the lower concentration (**Figure 3B**). At 4h pio, a higher concentration of rT3 and ST3 resulted in fewer tri-radiate spicules/embryo and at 8h pio, a higher concentration of rT3 resulted in more tri-radiate spicules/embryo compared to the lower concentration (**Figure 3B**).

When analyzing spicule length, TH treatment at the high concentration (10^-7^M) resulted in a significant effect of treatment on the length of spicules/embryo when tested at 4h pio (n = 170, H_5_ = 14.43, p = 0.013, η_2_ = 0.057); (**Figure 3C**). At the low concentration (10^-9^M) a significant effect of treatment was found at 8h pio (n = 234, H_5_ = 17.63, p = 0.003, η_2_ = 0.055); (**Figure 3C**). The results of the *post-hoc* comparisons between treatment groups for all time points are summarized in **Supplementary Data Sheet 1**, **Supplementary Table S7**, and are indicated by letters on the graphs (**Figure 3C**). Specifically, we found that rT3 and T3 resulted in longer spicules/embryo at 4h pio (10^-7^M) compared to the control (**Figure 3C**). We also found that at 8h pio (10^-9^M), T4 resulted in longer spicules/embryo compared to ST4 and ST3 (**Figure 3C**). The results of the dose-response analysis between concentrations for all time points are summarized in **Supplementary Data Sheet 1**, **Supplementary Table S6**, and indicated with asterisks on the graphs (**Supplementary Data Sheet 2**). Specifically, we found that at 4h pio a higher concentration of DMSO in the control resulted in shorter spicules/embryo compared to the lower concentration, but a higher concentration of rT3 resulted in longer spicules compared to the lower concentration (**Figure 3C**). At 8h pio, a higher concentration of ST4 and ST3 resulted in longer spicules/embryo and at 12h pio, a higher concentration of ST4 and T3 resulted in longer spicules/embryo compared to the lower concentration (**Figure 3C**).

**Figure 4:**
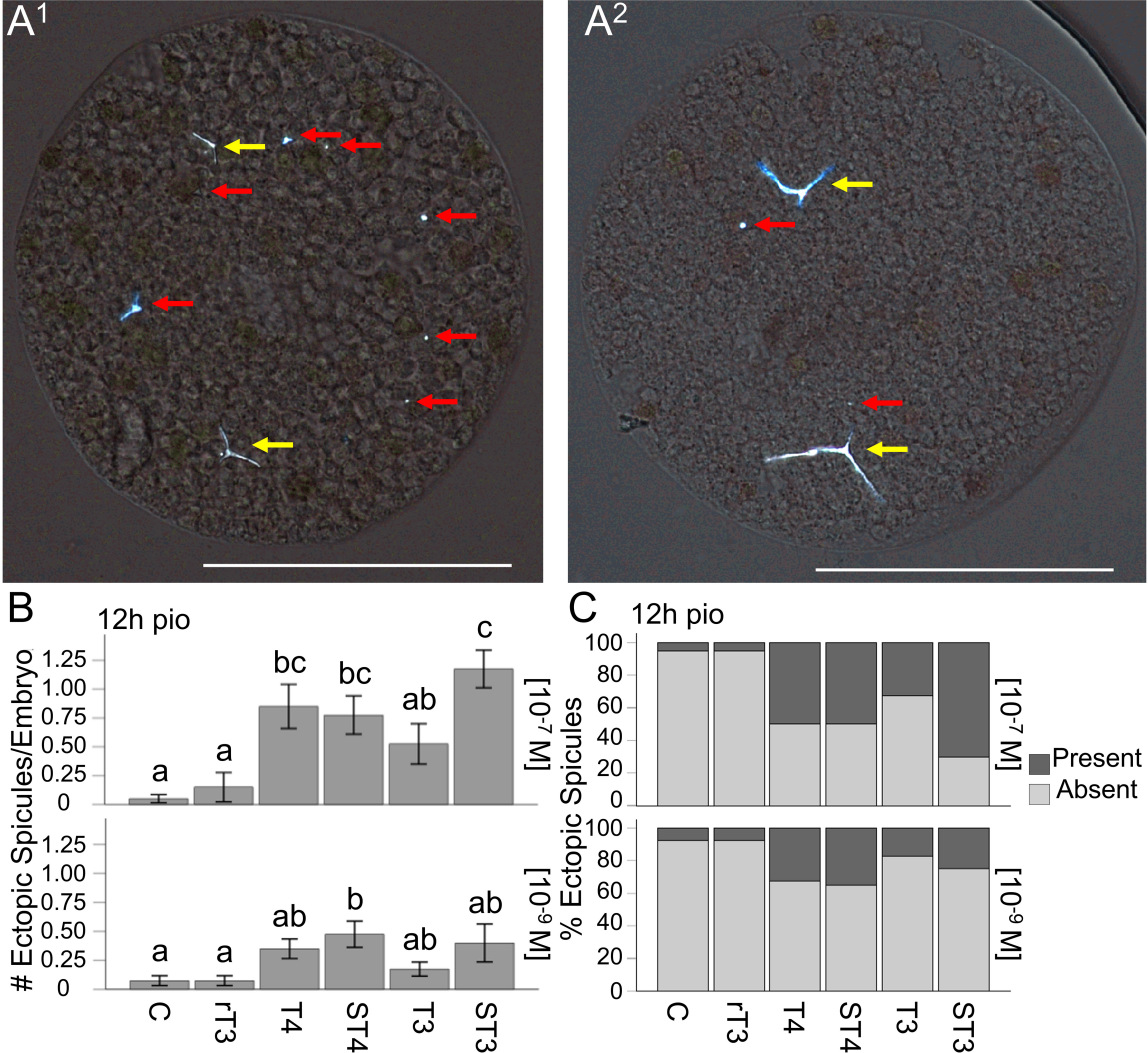
STH treatment results in the increased formation of ectopic spicules. **(A^1^)** Representative image of ectopic spicule patterning phenotypes, suggesting ectopic spicule formation by both skeletogenic and non-skeletogenic cells. (**A^2^**) Ectopic spicules forming alongside existing elongated triradiate spicules. **(B)** Number of ectopic spicules for all treatments at 12h pio, for both concentrations. **(C)** Ectopic spicule presence for all treatments at 12h pio, for both concentrations. The red arrows indicate ectopic spicules, and the yellow arrow indicates primary spicules. White scale bar represents 100µm, error bars represent ± 1 unit se. Letters above bars represent significant differences between treatments (p<0.05). Statistical comparisons are summarized in **Supplementary Data Sheet 1**.

We tested TH effects on ectopic spicule development (see **Figure 4A^1-2^** for representative images; **Figure 4** - # of ectopic spicules/embryo and presence of ectopic spicules) at four discrete time points starting from the initial point of observation (pio). TH treatment at both concentrations resulted in a significant effect of treatment on the number of ectopic spicules/embryo when tested at 12h pio (H_5_ = 60.30, p <0.001, η_2_ = 0.24, and H_5_ = 18.44, p = 0.002, η_2_ = 0.057, respectively); (**Figure 4B**). The results of the *post-hoc* comparisons between treatment groups for all time points are summarized in **Supplementary Data Sheet 1**, **Supplementary Table S8a**, and indicated with letters on the graphs (**Figure 4B**). Specifically, we found that T4, ST4, and ST3 resulted in more ectopic spicules/embryo compared to the control and rT3, and ST3 also resulted in more ectopic spicules/embryo than T3 at 12h pio (10^-7^M); (**Figure 4B**). We also found that at the lower concentration, ST4 resulted in more ectopic spicules/embryo than the control and rT3. The results of the dose-response analysis between concentrations for all time points are summarized in **Supplementary Data Sheet 1**, **Supplementary Table S9**, and indicated with asterisks on the graphs (**Supplementary Data Sheet 2**). Specifically, we found that at 0h pio at the higher concentration of rT3 resulted in fewer ectopic spicules/embryo compared to the lower concentration. At 4h pio, a higher concentration of T3 resulted in fewer ectopic spicules/embryo and at 12h pio, a higher concentration of T4 and ST3 resulted in more ectopic spicules/embryo compared to the lower concentration.

Similarly, TH treatment at the high concentration (10^-7^M) resulted in a significant effect of treatment on the presence of ectopic spicules when tested at 12h pio for both concentrations (H_5_ = 60.61, p < 0.001, η_2_ = 0.24, and H_5_ = 17.41, p = 0.004, η_2_ = 0.053, respectively); (**Figure 4C**). The results of the *post-hoc* comparisons between treatment groups for all time points are summarized in **Supplementary Data Sheet 1**, **Supplementary Table S8b**, and indicated with letters on the graphs (**Figure 4C**). Specifically, we found that T4, ST4, and ST3 resulted in increased presence of ectopic spicules/embryo compared to the control and rT3, and ST3 also resulted in increased presence of ectopic spicules/embryo than T3 at 12h pio (10^-7^M) (**Figure 4C**). The results of the dose-response analysis between concentrations for all time points are summarized in **Supplementary Data Sheet 1**, **Supplementary Table S9**, and indicated with asterisks on the graphs (**Supplementary Data Sheet 2**). Specifically, we found that at 0h pio a higher concentration of rT3 resulted in fewer embryos with ectopic spicules compared to the lower concentration. At 4h pio, a higher concentration of T3 resulted in fewer embryos with ectopic spicules and at 12h pio, a higher concentration of ST3 resulted in more embryos with ectopic spicules compared to the lower concentration.

In addition to these quantitative effects, we also observed that in embryos with many ectopic spicules, the ectopic spicules were frequently not located next to primary spicules and occasionally formed a distinct ring around the interior of the embryo. This geometry roughly aligns with the syncytial ring of PMCs that typically do not develop spicules outside of the two ventrolateral clusters along this ring (**Figure 4A^1^**). We also found that disproportionally small early spicules were located next to pigment cells. We did not analyze these phenotypes as we were unable to ensure that flattening of the embryos did not change the relative location of cells and spicules.

### 3.2 *Strongylocentrotus purpuratus* integrin αPβG shows comparable structure to human αvβ3 and binds STHs with higher affinity that non-sulfated ligands in silico.

We analyzed the structure of the putative integrin thyroid hormone receptor in *S. purpuratus* (αPβG) and compared it to the human structure of the αvβ3 integrin, which has been shown to bind thyroxine and sulfated THs (12). The sea urchin genome features four possible orthologs of the human αv subunit, based on OMA browser analysis (https://omabrowser.org/oma/home/). Detailed sequence comparisons revealed the following percent similarity with the human sequence: 33.5% (Q9U6S1, e = 2.2e^-170^), 32.3% (A0A7M7T1D7, e = 6.1e^-125^), 27.7% (A0A7M7T120, e = 9e^-93^), and 27.2% (A0A7M7NI58, e = 2e^-61^). Based on sequence similarity and supported orthology searches, αP (Q9U6S1) therefore represents the most likely candidate for the α subunit ortholog. *S. purpuratus* does not have a subunit annotated as β3 and OMA browser analysis did not identify any orthologs for the human β3 subunit in the *S. purpuratus* genome. Still a Blast search revealed that βG (P92163) is the most similar sequence with a sequence similarity of 41.3% (e = 0). Reversing the search using the *S. purpuratus* βG subunit, the OMA browser identified one ortholog to this protein in humans, integrin subunit β1 (P05556), which has never been implicated in TH binding.

**Figure 5:**
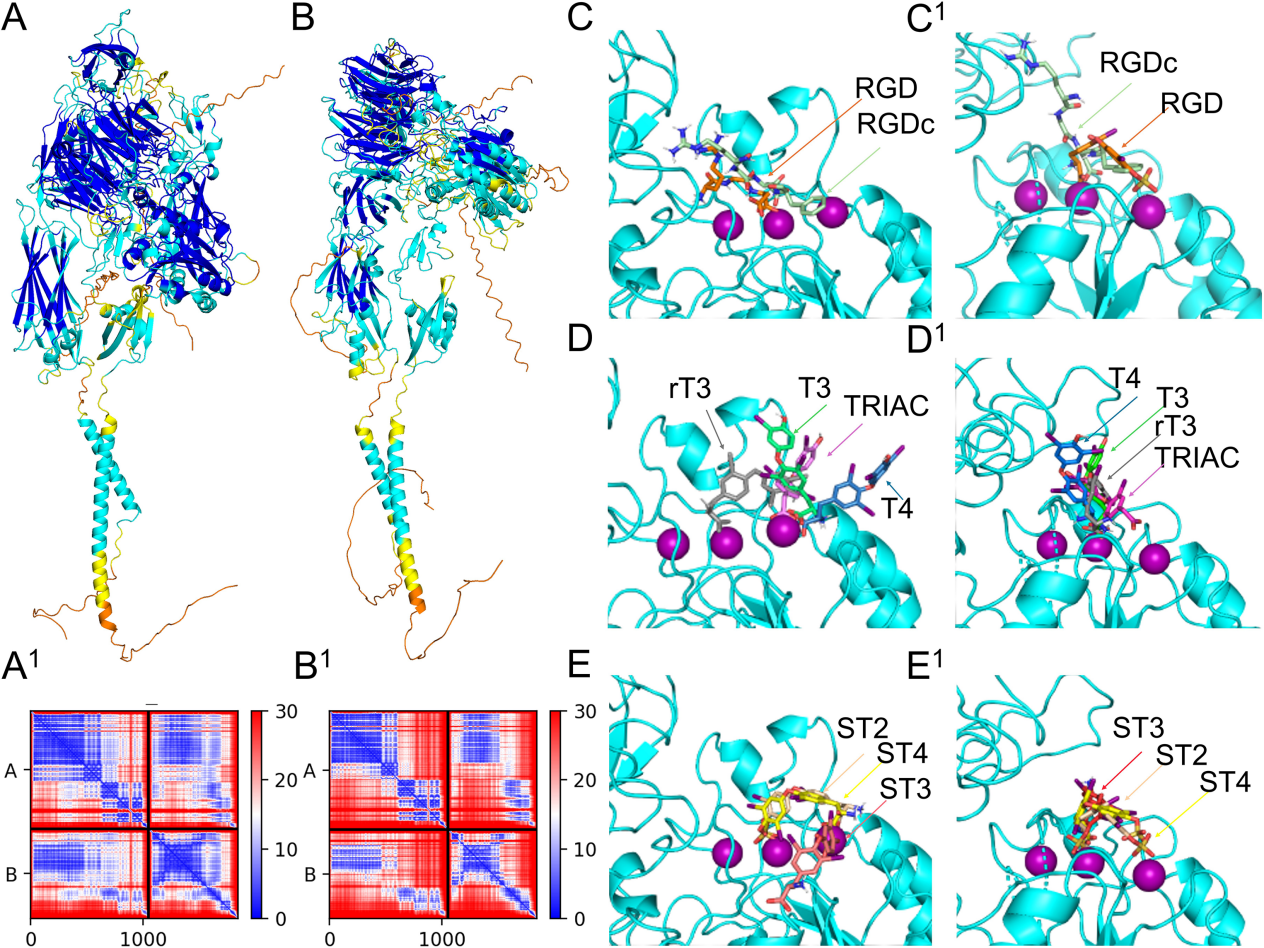
Assessment of integrin dimer receptor structure and binding locations of TH metabolites. *Homo sapiens* αvβ3 (P06756+P05106) predicted 3D structure colored by pLDDT score oriented with the α subunit on the left **(A)** and predicted aligned error plot **(A^1^)**. *Strongylocentrotus purpuratus* αPβG (Q9U6S1+P92163) predicted 3D structure colored by pLDDT score oriented with the α subunit on the left **(B)** and predicted aligned error plot **(B^1^)**. Differential binding affinity of RGD and RGDc between *Homo sapiens* integrin αvβ3 **(C)** and αPβG **(C^1^)**. Differential binding affinity of T4, T3, rT3, and TRIAC between *Homo sapiens* integrin αvβ3 **(D)** and αPβG **(D^1^)**. Differential binding affinity of ST4, ST3 and ST2 between *Homo sapiens* integrin αvβ3 **(E)** and αPβG **(E^1^)**.

The human αvβ3 structure was predicted accurately, with a pLDDT score of 81.1, pTM score of 0.746, and ipTM score of 0.741, and high local pLDDT scores, especially within the intercellular domain (**Figures 5A, A^1^**). Alignment of the intercellular domain of the *H. sapiens* αvβ3 integrin-predicted structure to 1L5G *H. sapiens* αvβ3 integrin crystal structure with RGD bound had a MatchAlign score of 3998.5 and an RMSD of 0.803, and the predicted structure cavity was very similar to that of the crystal structure. This binding pocket, which overlaps with the predicted TH binding pockets, was able to accommodate RGD without any residue conflicts that would limit RGD or TH binding (**Figure 5C**).

The *S. purpuratus* αPβG predicted structure also resulted in a high quality model, with a LDDT score of 76.9, pTM score of 0.644, and ipTM score of 0.62, local pLDDT scores, and pae scores (**Figures 5B, B^1^**). Of the four residues proposed to interact with THs in the αv subunit, three were conserved between *H. sapiens* and *S. purpuratus* αP, the remaining residue was not functionally similar (**Supplementary Data Sheet 3**, **Supplementary Figure S3.1**). Of the 10 residues proposed to interact with THs in the β3 subunit, six residues were conserved between *H. sapiens* and *S. purpuratus* βG*;* the remaining residues were not functionally similar (**Supplementary Data Sheet 3**, **Supplementary Figure S3.1**). Similarly to human αvβ3, RGD the binding pocket of αPβG accommodated RGD without any residue conflict (**Figure 5C^1^**), however RGD bound in slightly different poses between the human and sea urchin receptors (**Figure 5C, C^1^**). Details on the accuracy of the *in silico* binding approach for *H. sapiens* αvβ3 is shown in **Supplementary Data Sheet 3**, **Supplementary Figure S3.2**

While HADDOCK scores are not direct measures of binding affinity, they do provide confidence in the binding poses generated. Therefore, the pose generated with the highest HADDOCK score was used to predict binding affinity. HADDOCK scores for both, the human and sea urchin integrin receptors predicted binding to TH metabolites in the highest scoring pose and with all predictions reflecting favorable binding or negative energy used by the predicted binding. Both the human and sea urchin integrin receptors demonstrated the capacity to bind THs, with preferential affinity for different metabolites. **Figures 5C–E** visualizes the binding locations for specific ligands, including RGD and RGDc on human αvβ3. **Figures 5C^1^–E^1^** visualizes the binding locations for specific ligands, including RGD and RGDc on sea urchin αPβG. **Table 1** provides the HADDOCK scores and the estimated binding affinities for human and sea urchin receptors. We found that for the human αvβ3 integrin receptor, ST4 bound with the highest affinity, followed by ST3, TRIAC, ST2, T4, T3, and rT3 (**Table 1**, **Figures 5D, E**). For the sea urchin receptor, we found that ST4 also had the highest predicted affinity, followed by ST2, ST3, TRIAC, T4, T3, and then rT3 (**Table 1**, **Figures 5C^1^, E^1^**). Non-sulfated TH metabolites were predicted in similar poses as sulfated metabolites (**Figures 5D, D^1^**), however, in STH models, the orientation of the ligand was rotated so that the sulfate group bound to a Mg ion, forming a stronger bond that increased predicted binding affinity (**Figures 5E, E^1^**).

**Table 1:** Sulfated thyroid hormones have the highest predicted binding affinity of thyroid hormone metabolites to integrin αvβ3 from *Homo sapiens* and αPβG from *Strongylocentrotus purpuratus*.

Receptor	Ligand	HADDOCK score	Binding affinity ΔG
H. sapiens αvβ3	ST4	-68.5	-12.71
	ST3	-48.4	-10.83
	TRIAC	-50.7	-10.42
	ST2	-69.8	-10.29
	T4	-43.5	-9.73
	T3	-43.9	-9.61
	rT3	-43.5	-9.31
S. purpuratus αPβG	ST4	-61.5	-12.59
	ST2	-60.2	-11.58
	ST3	-52.4	-11.13
	TRIAC	-49.3	-10.56
	T4	-44.5	-9.21
	T3	-44.6	-9.08
	rT3	-41.8	-9.07

Receptor structures were predicted using AlphFold2 and docking used the HADDOCK webserver and PRODIGY-Ligand. Predicted ligand binding affinity is arranged from the strongest affinity to the weakest affinity for each species.

### 3.3 *Strongylocentrotus purpuratus* NTHR shows divergent structure from human orthologue and binds THs and STHs with low affinity

To assess the potential binding capacity of sulfated and non-sulfated ligands to the sea urchin NTHR, we also modeled the THRs from sea urchins and humans in combination with the dimerization partner RXR and analyzed the binding of TH metabolites, using AlphFold2 and CBDock2. To gain better insight into the quality of these models from an evolutionary perspective, we also analyzed THRs and their binding affinity to TH metabolites for the zebrafish *Danio rerio*, from the basal chordates *Ciona intestinalis* and *Branchiostoma floridae* and the mollusk *Crassostrea gigas* (see **Supplementary Data Sheet 4**, **Supplementary Figure S4.1** for alignment of these NTHRs).

**Figure 6:**
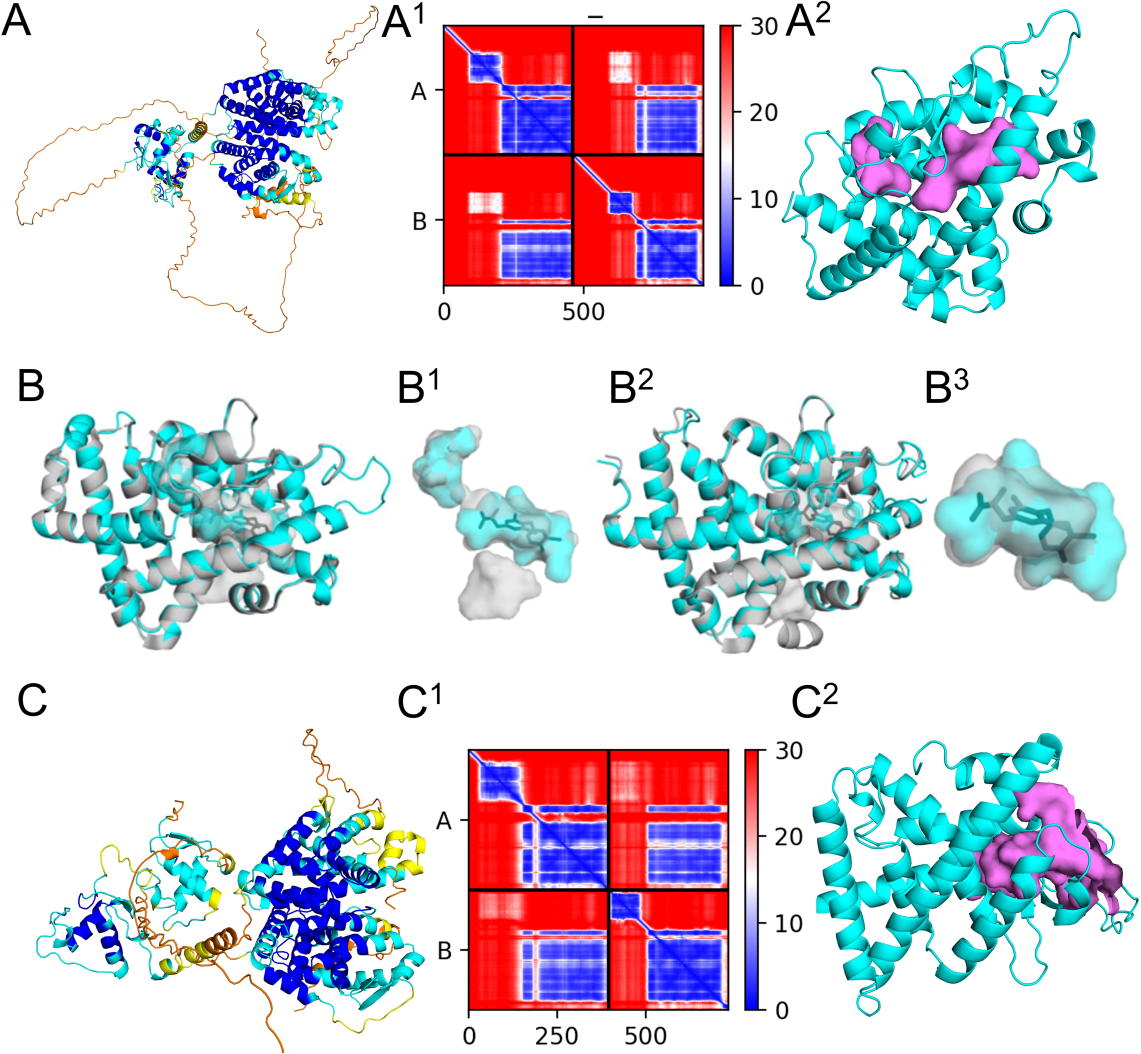
Modeling of human and sea urchin nuclear thyroid hormone receptor (NTHR) and retinoic X receptor (RXR) with binding location assessment of T3. Alphafold2 predicted thyroid hormone receptor beta (THRβ) biding pocket models align well with known crystal structures and show no conflicts between structural binding pockets or cavities and ligand positions in *Homo sapiens*. **(A)** *Homo sapiens* THRβ (P10828) and RXRα (P19793) predicted 3D structure complex oriented with the DNA binding domain (left) and the ligand binding domain (right), colored by pLDDT score, **(A^1^)** and the corresponding predicted aligned error plot **(A^2^)** THRβ from the predicted dimeric structure in cyan and large binding pockets in violet. **(B)** Ligand binding domain of THRβ predicted monomer (P10828) aligned to monomeric crystal structure with T3 bound (PDB: 3gws) with cartoon views of structures with pockets shown in surface view. **(B^1^)** Surface view of internal pockets with T3. **(B^2^)** Ligand binding domain of the thyroid hormone receptor β predicted dimer (P10828 + RXRα P19793) aligned to dimeric crystal structure with T3 bound (PDB: 4zo1) with cartoon views of structures with pockets shown in surface view. **(B^3^)** Surface view of internal pockets of the dimer with T3. **(C)** *Strongylocentrotus purpuratus* THR (A0A7M7N2J7) + RXR (A0A7M7PMF3) predicted 3D structure complex oriented with the DNA binding domain (left) and the ligand binding domain (right), colored by pLDDT score, **(C^1^)**and the corresponding predicted aligned error plot **(C^2^)** THRβ from the predicted dimeric structure in cyan and large binding pockets in violet.

We validated our modeling and metabolite binding approach, using the human monomeric thyroid hormone receptor beta (THRβ) and dimeric complex with retinoic x receptor alpha (RXRα), its binding pocket and its main ligand T3 (**Figures 6A, B**, **Supplementary Data Sheet 4**, **Supplementary Table S4.1** for model scores). The ligand binding domain of the predicted THRβ monomer (P10828) and heterodimer with RXRα (P10828 + RXRα P19793) aligned accurately to the monomeric crystal structure with T3 bound (PDB: 3gws). Alignment of the ligand binding domain of the *H. sapiens* predicted THR structure to 3GWS monomeric structure of the *H. sapiens* THR with T3 bound had a MatchAlign score of 1250.5 and an RMSD of 0.373. Visual inspection of the binding pocket shows that the predicted structure was very close to that of the crystal structure, and there were no conflicts between residues in the binding pocket and the position of T3 (**Figure 6B**). Similarly, the alignment of the ligand binding domain of the *H. sapiens* dimeric predicted structure in complex with RXR to 4ZO1 dimeric structure of the *H. sapiens* THR with RXR and T3 bound had a MatchAlign score of 1261.5 and an RMSD of 0.441. The monomeric structure of THR featured a fully enclosed binding pocket with a volume of 506 Å³, whereas the dimeric structure exhibited a slightly larger yet similarly enclosed pocket of 574 Å³. The binding pocket was very similar between these structures, and T3 was able to fit within the pocket of the predicted structure without conflicting with any of the residues (**Figures 6A, B**).

We then tested TH metabolite binding to the human THR heterodimer (**Table 2**, **Supplementary Table S4.2**). T3 exhibited the highest predicted affinity, followed by TRIAC. T3 interacted with five of the previously identified residues and three residues not identified by literature (Ile276, Ala279, Met313, Arg316, Arg320, Leu330, Asn331, Leu346, and His435) but did not interact with Leu341 or Phe455. Comparatively, T4 interacted with seven of the previously identified residues and four additional residues (Phe272, Ile276, Ala279, Met313, Arg316, Arg320, Leu330, Asn331, Gly344, Leu346, and Met442) but resulted in a lower predicted affinity. The lowest predicted affinity was observed for ST4, while ST3 showed intermediate binding affinity, ranking between TRIAC and T4 (**Table 2**, **Supplementary Table S4.2**). Dimerization with RXR generally increased binding affinity in our model, except for the biologically inert rT3, indicating greater predictive accuracy for active thyroid hormone interactions.

**Table 2:** Predicted CBDock2 binding affinity of thyroid hormone metabolites to thyroid hormone receptor (THR) monomeric and dimerinc (THR + RXR) for *Homo sapiens* and *Strongylocentrotus purpuratus*.

Receptor	T4	T3	rT3	TRIAC	ST4	ST3
*H. sapiens* THRβ	-6.5	-8.1	-7.8	-7.9	-3.6	-6.4
*H. sapiens* THRβ + RXRα	-7.1	-9.5	-6.8	-9	-5.3	-7.9
*S. purpuratus* THR	76.2	63	54.5	56.7	71.5	54.4
*S. purpuratus* THR + RXR cavity-detected	-6.5	-6.5	-6.6	-6.2	-6.1	-6
*S. purpuratus* THR + RXR template-based	1.6	-2.2	-2.7	0.4	4.1	1.7

Receptor structures were predicted using AlphFold2 and docking used CBDock2.

The *S. purpuratus* THRβ is the top BLAST result for the human THRβ in the sea urchin genome and shares 49.3% of the same amino acid sequence. This is higher compared to the other isoform in humans, THRα, with 46.7% sequence similarity. The RXR found in *S. purpuratus* is more similar in sequence to RXRα (79%) than to RXRβ (74.6%) or RXRγ (74.5%) and was thus the human receptor used for comparisons with other species. Modeling of the sea urchin THR monomer and dimer with RXR resulted in high pLDDT, pTM, ipTM scores, high local pLDDT scores (**Figure 6B^1^**), and pae scores (**Figure 6C**, **Supplementary Table S4.1**) and the predicted *S. purpuratus* THR dimeric structure was of high quality. CBDock2 did not detect a specific binding cavity analogous to that of *H. sapiens* in the monomeric structure among the top 20 cavities. Instead, a large cavity was present in the same general region of the protein upon visual inspection using Pymol. In the dimeric structure, CBDock2 successfully identified a cavity in this region of the protein, but it was substantially larger (3141 Å³) and open to the surface at multiple points (**Figure 6C**). Interestingly, in *H. sapiens* and *S. purpuratus*, CBDock2 also identified a binding cavity in the RXR receptor of the dimeric models. This cavity was 2734 Å^3^ in *H. sapiens* and 1051 Å^3^ in *S. purpuratus*, and both were slightly open to the outside of the protein. For the human receptor, T4 resulted in a binding affinity score of -7.4, while TETRAC and TRIAC resulted in scores of -7.3 (**Table 2**, **Supplementary Table S4.2**). For the sea urchin receptor, T4 resulted in a score of -6.6, TETRAC resulted in a score of -6.2, and TRIAC resulted in a score of -7.4 (**Table 2**, **Supplementary Table S4.2**).

We also analyzed the structures of several other THRβ proteins, to better understand the performance of our modeling approach for proteins without crystal structure and gain insight into structural changes in the THRβ across animal groups. The results of this analysis are presented in **Supplementary Data Sheet 4**, **Supplementary Figure S4.2**-**Supplementary Figure S4.7**. The *D. rerio* RXRαα has 87% sequence similarity with the human RXRα receptor. The *B. floridae* RXR has 67.4% sequence similarity with the human RXRα receptor. The *C. intestinalis* RXR has 61.8% sequence similarity with the human RXRα receptor. The *C. gigas* RXRα has 67.2% sequence similarity with the human RXRα receptor. All of these receptors were identified as HOG orthologs (Hierarchical groups contain genes that descend from a single common ancestral gene within a given taxonomic range) to the human receptor by the OMA browser. Modeling scores for all receptors are shown in **Supplementary Table S4.1**. Apart from *C. intestinalis* (see below), pTM and ipTM scores are above the overall model confidence cutoff and further suggest that interdomain and interprotein regions have a strong negative effect on the overall pLDDT score but that the predictions within domains are similar to the true structure with the backbone and most side chains predicted correctly. The ipTM score, which is well above the cutoff in most receptors, increases confidence that RXR is positioned in the correct area of the THR structures, although there may be small errors in exact positioning. While some structures contained unstructured regions, due to the buried nature of the binding pocket and upon visual inspection, these regions did not conflict or alter binding affinity calculations. Therefore, no regions were removed from the predicted structures before docking.

None of the 12 residues previously identified to interact with TH metabolites directly within the binding pocket of the *H. sapiens* THR are conserved across all six model species (**Supplementary Figure S4.1**). However, only four of these residues are highly diversified across the species. One of these residues is conserved in all species except *C. intestinalis* and *C. gigas*, but the residue substitutions in these receptors are not functionally similar to those of the other species. Eight of these residues are different across many of the species, but the residues are all functionally similar in metrics like hydrophobicity and side chain size and may still be able to form bonds with TH ligands and explain differential binding of TH metabolites. Six of these eight residues are identical between *H. sapiens* and *S. purpuratus*. Mapping sequence conservation of THRs in the select species onto the *H. sapiens* predicted structure, nearly 18% of the residues in the structure were conserved across all species, and these residues were spread across all three domains (**Supplementary Figure S4.1**).

Binding pocket size for predicted THR models varied substantially across species, influencing predicted binding affinities (**Supplementary Figures S4.2**-**S4.5**). Similarly, both *C. gigas* and *C. intestinalis* exhibited surface-exposed cavities in both monomeric and dimeric states; however, CBDock2 did not rank these among its top 20 cavity predictions, suggesting lower confidence in their functional relevance. In *D. rerio*, the monomeric structure contained a binding pocket of 574 Å³, whereas dimerization resulted in a reduced pocket volume of 501 Å³. The *B. floridae* monomer had a comparatively smaller cavity of 349 Å³, which expanded significantly to 610 Å³ upon dimerization.

For all species examined, binding affinity predictions differed between monomeric and dimeric structures for THR and varied across ligands (**Supplementary Table S4.2**). In *D. rerio*, the effect on binding affinity of dimerization was reversed, resulting from the smaller predicted binding pocket of the dimeric structure that changed the positioning of certain residues important to binding. Dimerization reduced predicted binding affinity, likely due to the smaller binding pocket and altered positioning of key binding residues. In *B. floridae*, dimerization was essential for achieving negative binding affinity predictions, indicating that spontaneous ligand binding was only observed in the dimeric state. As expected, TRIAC was the highest-affinity ligand in *B. floridae*, though binding affinities were lower overall compared to vertebrates. Invertebrate binding affinity predictions were positive in most cases, suggesting it would require energy to result in binding instead of spontaneous reactions between the receptor and ligand.

As there were no identified cavities in *C. intestinalis* and *C. gigas* in the approximate location of the vertebrate binding pocket, only template-based docking scores are presented for these two receptors (**Supplementary Table S4.2**). In *C. gigas*, binding affinity scores indicate a lack of prospective binding. Although T3 and ST3 had negative affinity values, these are considerably weaker than the -6 to -9 range observed in other species. This suggests that any spontaneous binding is unlikely with the current structural model. Moreover, spontaneous binding was only detected in the monomer, while the dimer exhibited positive binding affinity values, indicating that, if the structural model is correct, THs do not bind with any biological relevance in the dimeric system key for vertebrate TH signaling. In *C. intestinalis*, all binding affinity values for the monomer and dimer were positive, indicating no spontaneous binding in this receptor, as this receptor functioned as a negative control.

## 4 Discussion

We tested the pharmacological effects of STHs on embryonic skeletogenesis in *S. purpuratus* to gain insight into the signaling mechanism of THs in sea urchin development. We then analyzed putative canonical and non-canonical TH receptors *in silico* for their ability to bind THs. Together our results support the hypothesis that embryonic skeletogenesis involves an integrin receptor in sea urchins.

### 4.1 STHs, active ligands of integrin receptors in mammals, accelerate skeleton formation and induce ectopic spicule formation in sea urchin embryos

Embryonic skeletogenesis occurs concurrently with gastrulation in *S. purpuratus*, and PMCs form a distinct ring around the invaginating vegetal plate, creating cytoplasmic cords with clusters of PMCs (48–50). These PMCs create endoskeletal spicules composed of calcite, magnesium, extracellular matrix, and occluded matrix proteins. The granules are rhombohedral initially, eventually extending into tri-radiate spicules that extend from the PMCs at the tips of each branch (48). The regulatory network underlying sea urchin skeletogenesis has been extensively studied (19, 51). VEGF signaling is required to initiate spicule formation and regulates key transcription factors from the skeletogenic gene regulatory network, Alx1 and Ets1 which are phosphorylated by MAPK (Röttinger et al., 2004). Recent work has shown that T4 and to a lesser extent T3 accelerate spicule formation during sea urchin gastrulation (11, 17, 22). T4 has also been shown to increase ERK1/2 activity (17, 22) and skeletogenic transcription factors are differentially expressed in response to TH treatment (11, 22). We previously hypothesized that an additional pathway functions in skeletogenic regulation, which involved an integrin receptor activating MAPK signaling and phosphorylating specific components of the skeletogenic gene regulatory network (11, 22). THs, specifically T4, can bind to the integrin dimer αvβ3 and activate MAPK (ERK1/2) activation in mammalian angiogenesis and a range of cancers (8, 12, 52–58). This signaling system also interacts with VEGF signaling and shows a high level of similarity with the sea urchin skeletogenesis signal transduction system (8, 10, 11, 22, 24, 52–57, 59, 60). Along with T4 and T3, STHs, specifically ST4 and ST3, had an acceleratory effect on spicule formation in our study. The finding that STHs show similar and in some cases even enhanced effects on skeletogenesis initiation as non-sulfated THs supports our hypothesis that the TH signal is mediated via an integrin receptor, since recent *in silico* docking studies suggest that sulfated TH ligands (ST2, ST3 and ST4) are predicted to have the highest binding affinities to integrin αvβ3 compared to RGD and a range of TH metabolites (12).

The STH effects on skeleton formation suggest that sulfated ligands may function as signaling molecules in sea urchin skeletogenesis, and may be higher affinity ligands than the previously tested T4, T3, TRIAC, TETRAC and T2. Still, we know little about how STHs are converted or metabolized in sea urchin embryos or the seawater, in which they are applied. Considering the prolonged exposure to these hormones in our experiments, we can therefore not exclude the possibility that different sulfated or non-sulfated metabolites may be binding to the receptor. We know however, that cells in proximity of endodermal neurons in the growing archenteron and mouth region contain thyroxine (17) and while endogenous levels of sulfated hormone and sulfotransferase activity has not been documented in sea urchin embryos, this is the first developmental evidence that exposure to STHs can elicit responses in an invertebrate and is the second in metazoans, with STHs restoring weight gain in hypothyroid rats (61). Generally, STHs are viewed as inactive ligands in mammals with important functions in fetal development and TH metabolism (62–65). Our findings presented here and the fact that STHs cannot bind to the NTHR (see below), suggests that these developmental effects are mediated via a non-canonical TH signaling pathway. Still, we were able to identify some important differences in developmental effects between different sulfated ligands. ST3 and ST4 had the strongest effects on the acceleration of spicules formation in our assay and our modeling data confirm that these ligands have the highest *in silico* binding affinity to the sea urchin integrin receptor candidate. In mammals and other vertebrates, T3 has been identified as the primary ligand for the genomic pathway and T4 is generally considered a pre-hormone. Still, other iodinated ligands have been identified as signaling molecules in other animal phyla. For example, TRIAC (triiodothyroactic acid) has been shown to be an active ligand of the nuclear thyroid hormone receptor in amphioxus, where the hormone regulates metamorphic development (66, 67). The fact that different TH ligands may be involved in the regulation of development in animals provides interesting insights into the evolution of TH signaling in animals. As these different ligands, which can be synthesized by a range of animal and non-animal groups (2, 3, 10, 16, 17, 24), can activate the same or different signal transduction pathways as in vertebrates, they may have acquired their function in development independently.

In contrast to spicule initiation, we did not observe consistent effects of THs on skeleton elongation in our study. At 4h pio we observed significantly elongated spicules for rT3 and T3 treatment and all THs resulted in slightly longer, albeit not significant, elongation 4h pio. At the lower concentration T4 resulted in significantly longer spicules 8h pio, but that trend did not continue 12h pio. If THs impacted the rate of elongation, we would expect to see a continuous increase in spicule length over time. We therefore interpret the slightly longer skeletons in the TH treatments 4 pio as a direct consequence of earlier spicule initiation in these treatments, rather than an effect on the spicule elongation process. This also aligns with previous work on TH effects on the larval skeleton, suggesting that hormones do not impact the length of skeletons until after rudiment development has started (22, 68). The fact that rT3 and T3 resulted in longer spicules at 4h pio, however could be explained by the differential interaction of these ligands with the integrin receptor. Tobi et al. (12), found that *in silico*, rT3 has a lower affinity to αvβ3 than T3 and binds to a similar location in the protein as T4. Interestingly, in mammalian cancers, rT3 and T4 have similar physiological effects on cancer proliferation via the integrin receptor (69), suggesting that the rT3 effects we observed in our study may also be mediated by the integrin receptor. Still, rT3 showed initially an inverse dose response on spicule elongation, with the lower concentration resulting in longer spicules than the higher concentration at 4h pio. This inverse dose response disappeared at later time points, suggesting that rT3 may activate more than one pathway after initial exposure.

Of the THs tested, ST4 and ST3 had the strongest effects on ectopic spicule formation, with dose-dependent responses of number of spicules, number of ectopic spicules, and presence of ectopic spicules. Although rare, it is well documented that additional (more than two) spicules can be formed during normal sea urchin skeletogenesis (48). While it remains unknown whether there is a function of ectopic spicules in normal development, previous work also noted the effects of THs on ectopic spicule formation. For example, Taylor & Heyland (22) reported additional early and tri-radiate spicules in response to T4 and to a lesser extent T3 treatment. This work also reported protrusions of the skeletal arms and duplicate arms, neither of which were observed in the current study, as our observations did not extend past 12h pio. With the predicted higher binding affinity of STHs in the sea urchin integrin receptor based on our modeling work, the formation of ectopic spicules in the STH treatment could provide insight into the signal transduction mechanism of THs via the integrin pathway. Specifically, it will be a critical phenotypic response to THs, to further analyze the interaction between VEGF and integrin signaling (see also above for discussion).

In addition to STHs, rT3, T4, and T3 also resulted in higher rates of ectopic spicule formation than DMSO controls, although less than those of STHs and more inconsistent across time points and concentrations. Our experimental design did not allow for a precise localization of ectopic spicules, as larvae were flattened under a cover slide before imaging and it is possible that spicule location may have shifted in this process. Despite this, we believe that the ectopic spicules in our experiment primarily originated from the region of the syncytial ring of skeletal primary mesenchyme cells, as we found that ectopic spicules were frequently located in a ring like arrangement in the area of where skeletal primary mesenchyme cells in sea urchin embryos are located in relation to the archenteron. Based on our model of integrin mediated TH signaling, key transcription factors in the skeletal gene regulatory network such as Alx1 and Ets1 are phosphorylated. Alx1 plays a central role in the differentiation of PMCs and upregulates the expression of biomineralization genes (70). Alx1 has also been shown to positively regulate VEGF receptor (19, 71), a necessary component of the sea urchin skeletogenic gene regulatory network. Importantly, overexpression of VEGF has been shown to cause additional aggregates of PMCs beyond the two ventrolateral clusters typical of development which then produced ectopic spicules (50), and both, VEGF and TH signaling may function through similar MAPK-dependent pathways (20, 22, 50). While THs may be directly causing formation of ectopic spicules by activating VEGF signaling via Alx1 upregulation, it is also possible that they are indirectly affecting the timing of localization of PMC migration and thus leading to additional spicules in locations where they typically do not form.

### 4.2 Modeling of the sea urchin integrin receptor supports a role of STHs and non-sulfated THs as active ligands to the non-canonical pathway

Based on current evidence, sea urchin TH signaling appears to be at least partially regulated by a membrane‐bound receptor that is inhibited by RGD peptides, strongly suggesting the involvement of an integrin dimer—similar to that observed in humans (11, 22). The sea urchin receptor designated αP is orthologous to the human integrin αv subunit, however, no ortholog for the human β3 subunit was identified. Instead, the closest BLAST result corresponds to the sea urchin βG subunit, which is orthologous to human β1.

In humans, integrin αvβ3 is well documented to activate MAPK phosphorylation upon TH binding (8, 12, 52, 53, 55–57, 72). Although the human β1 subunit has not been directly linked to TH signaling, it can independently activate MAPK pathways with downstream effects on cell migration and apoptosis when associated with collagen (73). Human integrin αv is not known to pair with β1 and form a heterodimer capable of generating an RGD recognition site (74), even though β1 does contain metal ion–dependent adhesion sites that are critical for ligand binding. For the human receptor, T3 was found to bind more closely to the RGD binding site than T4, as previously reported by Tobi et al. (12), although this difference in position was not strong enough to suggest two distinct binding sites within these two conformations that was suggested by Lin et al. (31). Instead, Cody et al. (53), suggested that the size of the TH ligands allows them to bind at the same site as RGD, which appears to be what our models predict. This also explains the greater number of interactions with the β subunit compared to the α subunit.

A lack of β subunit conservation between humans and sea urchins suggests that this group has diversified greatly in different taxa. However, the predicted binding affinity of the sea urchin αPβG suggests that this dimer may be able to bind THs, including STHs with a high binding affinity. While testing has been limited, STHs may be active ligands for the TH-binding integrin receptor in sea urchins and thus should be further studied regarding their formation, metabolism, transport, and binding to determine if they play active biological roles instead of or in addition to known ligands. As in humans, the sea urchin predicted binding affinity is greater for T4 compared to T3, which supports the findings that T4 has greater developmental effects on sea urchins (11, 17, 22). However, there is not enough information currently available to verify the sea urchin subunits, but based on docking scores, STHs show a strong potential to be active ligands that bind to the sea urchin integrin.

Known heterodimer partners of αv include β3, β5, β6, and β8, of which there are no orthologs in sea urchins (74). Predicted binding affinities and single-cell expression data indicate that the sea urchin βG subunit is highly expressed during early developmental stages when sea urchins are sensitive to THs (75, 76). Future work can assess the *in silico* dimerization potential and binding affinity of these subunits with αv. It can also focus on testing binding affinities of integrin dimers in heterologous expression systems and explore *in vitro* and *in vivo* binding assays of ligands in sea urchin development. Pharmacological studies should also include a broader range of ligands, such as ST2, which appears to have a high affinity to the integrin receptor but was not tested in this study.

### 4.3 Modeling of the sea urchin nuclear TH receptor provides little evidence for TH binding

While sea urchins have the receptors from both known TH signaling pathways, it is unclear if the sea urchin THRβ receptor bind THs (5). Our modeling of the sea urchin THRβ shows a large cavity in the same region as the vertebrate TH binding pocket and docking was unable to recover differential binding of different TH metabolites. This suggests that THs do not bind in this pocket as it is not conserved and is not close to the surface of the protein. Docking revealed that in the sea urchin models, there was stronger binding affinity of TH metabolites in a binding pocket in RXR which may explain binding if the sea urchin THRβ does not directly bind THs, although this requires further investigation both *in vitro* or *in vivo*. A surprising finding was that ST3 had a higher binding affinity than T4 in the human receptor. STHs have never been shown to bind to NTHRs, but this may be partially due to lack of studies. ST3 was able to fit within the predicted binding pocket, although with a lower affinity than the active metabolites T3 and TRIAC. If STHs could be used as an indicator of non-genomic signaling, attempts should be made to assess the binding affinity of ST3 to the NTHR in order to determine if this finding was a result of mis-predicted side chains that allowed the sulfate group of ST3 to fit into the binding pocket or if ST3 can effectively bind to this receptor. It is also possible that regardless of true binding affinity, the rapid degradation of STHs in conjunction with transport limitations results in no biologically relevant STH concentrations that can be maintained and signal in the nucleus. While STH specific transport has been proposed for the plasma membrane (77) we lack information on how STHs could be effectively transported into the nucleus. Binding of ST3 should also be tested *in vitro* or *in vivo*, at least in the human receptor, to test if this metabolite is truly inert before further assumptions about its inactivity result in setbacks in understanding TH metabolite functions.

Docking analyses revealed differences in the predicted binding profiles of TH ligands between vertebrate and invertebrate receptors. In both *H. sapiens* and *D. rerio* NTHRs, T3 and TRIAC were the top-ranking ligands. This ranking is consistent with previous modeling work (78). However, experimental binding affinities were reversed with T3 having a binding affinity of -12.6 kcal/mol (79) and TRIAC with a stronger affinity of -14.04 kcal/mol (80). In our docking work, different metabolites interacted with slightly different subsets of binding pocket residues and these identified residues were largely consistent with residues identified in a modeling study of the rat NTHR (81) and crystal structures of the LBD with different THs bound (37, 47). Compared to 3GWS, the predicted T3 position lacked bonds between R282, M310, and A317 and T3 (37). This may explain slightly lower predicted values than those found in the literature, as some residues were not flexible enough in the modeled structure to be within binding distance estimated by CBDock2.

In contrast to the human receptor, the binding patterns observed in other species varied considerably. In *D. rerio*, after T3 and TRIAC, rT3 ranked higher than other metabolites, suggesting potential differences in receptor-ligand interactions compared to humans or difficulty with the smaller rT3 ligand of the docking software. In *B. floridae*, TRIAC ranked as the top ligand as expected based on earlier studies (66, 67) but the large binding pocket in these models resulted in minimal score differences among ligands, indicating that the pocket may be too large to allow for fine discrimination in binding affinities, either naturally or because of incorrect modeling. Models for *Ciona intestinalis* and *Crassostrea gigas* did not demonstrate biologically relevant TH binding, which may reflect evolutionary divergence in receptor structure and align with previously published work, suggesting that THs are not able to bind to these nuclear hormone receptors (NHRs) (5). In sea urchin models, rT3 ranked highest despite very similar scores among ligands, which again suggests that the large binding pocket does not support strong, differentiable interactions.

The human and sea urchin models frequently positioned the ligand within a binding cavity in RXR. Gellrich et al. (82), found that T4, TETRAC, and TRIAC can bind to RXR in humans. Predicted binding affinities are similar or better in the sea urchin receptor than in the NTHR and RXR may provide a possible binding location if the binding pocket in the NTHR is not selective enough for specific metabolites. TH ligands have relatively similar sizes and weights and in NTHRs the binding affinity is specifically determined by the number of iodines (83). Further investigation is required to understand the potential role of RXR as a T4 receptor in sea urchins, as it could provide an additional explanation for the stronger developmental action of T4 over T3 in invertebrates compared to vertebrates (5).

## Data Availability

The original contributions presented in the study are included in the article/**Supplementaryary Material**. Further inquiries can be directed to the corresponding author.
