# Supplementary material for: Developmental effects of sulfated thyroid hormones in sea urchin skeletogenesis suggest activation of non-canonical thyroid hormone signaling pathway
Source: Front Endocrinol (Lausanne). 2025 Aug 21;16:1648899. doi: 10.3389/fendo.2025.1648899 (PMC12408288; doi:10.3389/fendo.2025.1648899)
Supplement: Supplementary file 2 [file DataSheet2.docx]

**

**

**Supplement 2: Thyroid hormone exposure effects on skeletogenesis are dose-dependent in *Strongylocentrotus purpuratus* embryos.** Embryos were exposed to thyroid hormone metabolites and monitored until 50% spicule development in [10^-7^ M] T4 treatment (10.9 hpe = 0h pio) with the following three timepoints every 4h. The dataset was split by time and treatment and T-tests were used for statistical analysis. **(A)** Mean number of early spicules. **(B)** Mean number of spicules. **(C)** Mean skeletal stage. **(D)** Mean number of tri-radiate spicules. **(E)** Mean spicule length. **(F)** Mean number of ectopic spicules. **(G)** Presence of ectopic spicules. Error bars represent ± 1 unit se. *: p ≤ 0.05, **: p ≤ 0.01, ***: p ≤ 0.001, indicates significant difference from higher concentration.
