# Supplementary material for: Developmental effects of sulfated thyroid hormones in sea urchin skeletogenesis suggest activation of non-canonical thyroid hormone signaling pathway
Source: Front Endocrinol (Lausanne). 2025 Aug 21;16:1648899. doi: 10.3389/fendo.2025.1648899 (PMC12408288; doi:10.3389/fendo.2025.1648899)
Supplement: Supplementary file 3 [file DataSheet3.docx]

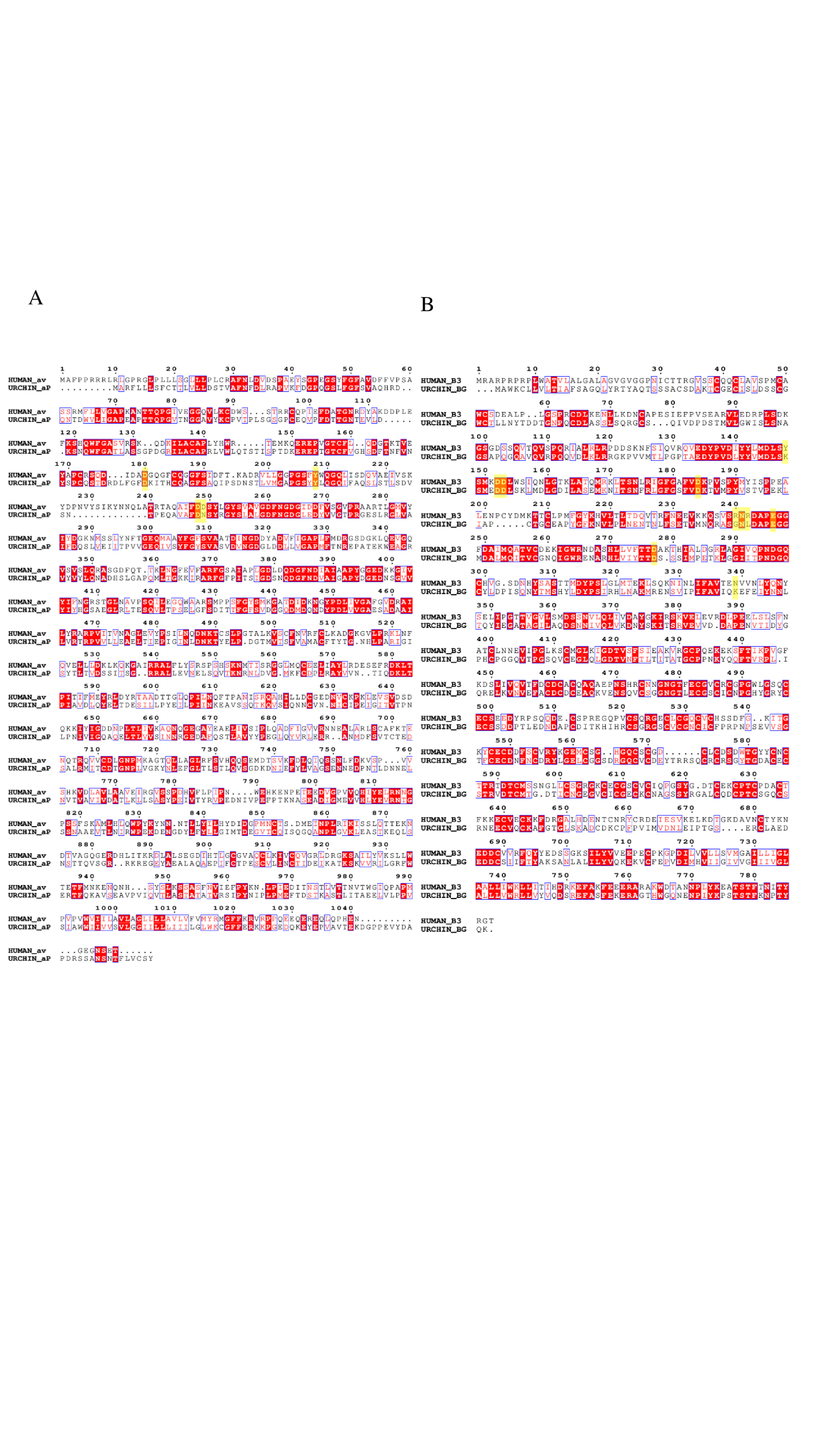


Figure S3.1. Three of four α subunit and six of ten β subunit residues predicted to bind thyroid hormones in the Homo sapiens αvβ3 integrin (P06756 + P05106) are conserved in the Strongylocentrotus purpuratus αP and βG integrin subunits. MSA generated using ClustalW for (A) α and (B) β subunits. Residues in red are conserved, blue boxes represent residue positions that differ but remain functionally similar, yellow shows residues previously predicted to bind thyroid hormones in the H. sapiens receptor based on previous docking work by Tobi et al. (2022).


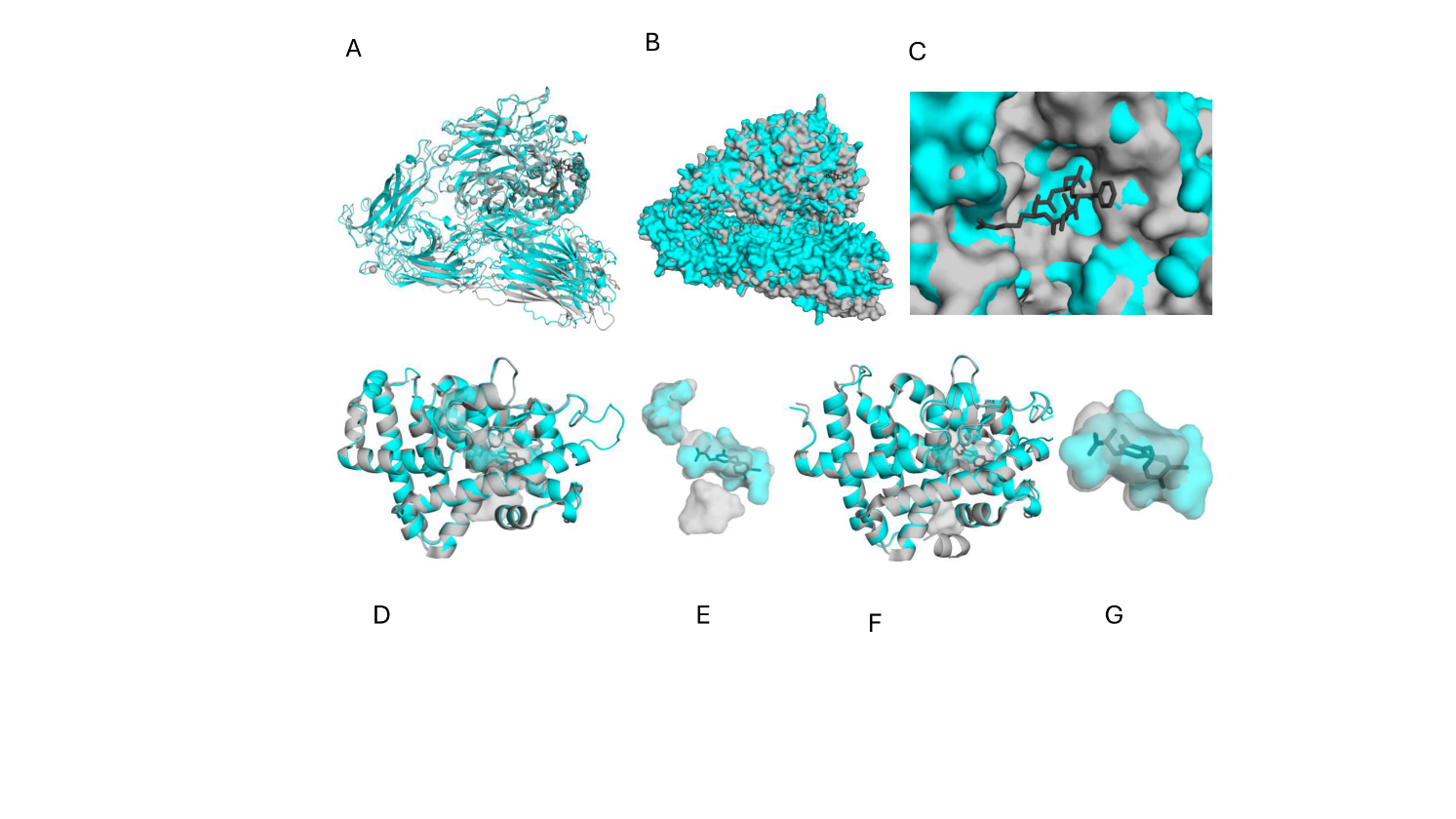


C)

B)

A)

**Figure S3.2. Alphafold2 predicted αvβ3 integrin receptor models align well with known crystal structures and show no conflicts between structural binding pockets or cavities and ligand positions in *Homo sapiens*.** **(A)** Intercellular domain of the predicted αvβ3 integrin (P06756 + P05106) in cyan aligned to crystal structure with cyclic RGD bound (PDB: 1L5G) with cartoon views of structures in grey. **(B)** Surface view of αvβ3 integrin with cyclic RGD bound (PDB: 1L5G). **(C)** Closer surface view centered on cyclic RGD binding site. Predicted structures shown in cyan, crystal structures shown in grey and ligands from crystal structures shown in black.
