# Supplementary material for: Developmental effects of sulfated thyroid hormones in sea urchin skeletogenesis suggest activation of non-canonical thyroid hormone signaling pathway
Source: Front Endocrinol (Lausanne). 2025 Aug 21;16:1648899. doi: 10.3389/fendo.2025.1648899 (PMC12408288; doi:10.3389/fendo.2025.1648899)
Supplement: Supplementary file 4 [file DataSheet4.docx]

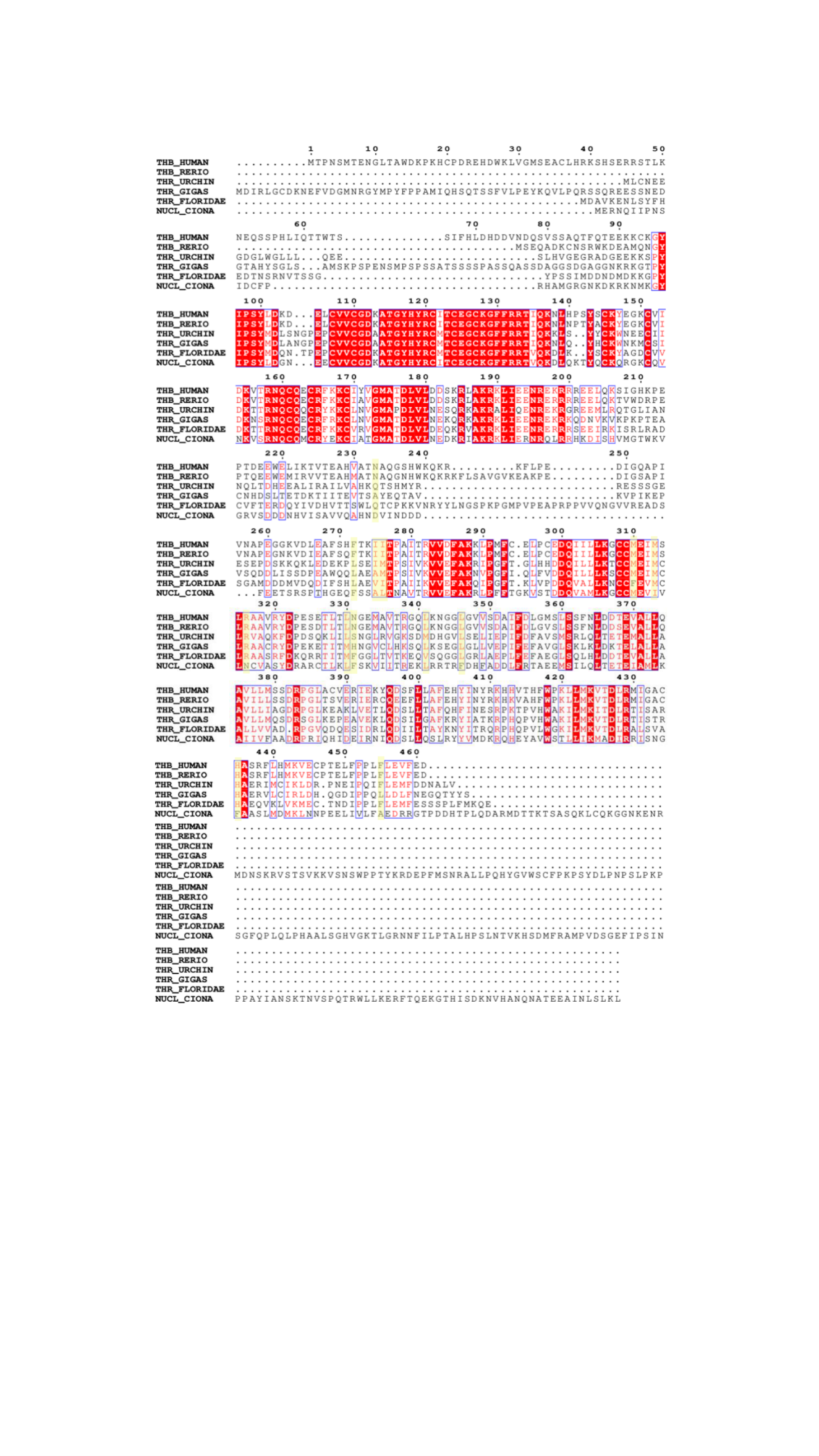


Figure S4.1. Many residues involved in the binding of thyroid hormones to the *Homo sapiens* thyroid hormone receptor β (P10828) are not well conserved. MSA includes *Danio rerio* thyroid hormone receptor β (Q9PVE4), *Strongylocentrotus* *purpuratus* thyroid hormone receptor β (A0A7M7N2J7), *Crassostrea gigas* thyroid hormone receptor (A0A0F6V015), *Branchiostoma floridae* thyroid hormone receptor (A7L5U9), *Ciona intestinalis* nuclear receptor (H2XS16). Residues in red are conserved in all included species, blue boxes represent residue positions that differ but remain functionally similar, yellow shows residues previously identified to bind thyroid hormones in the *H. sapiens* receptor.

**
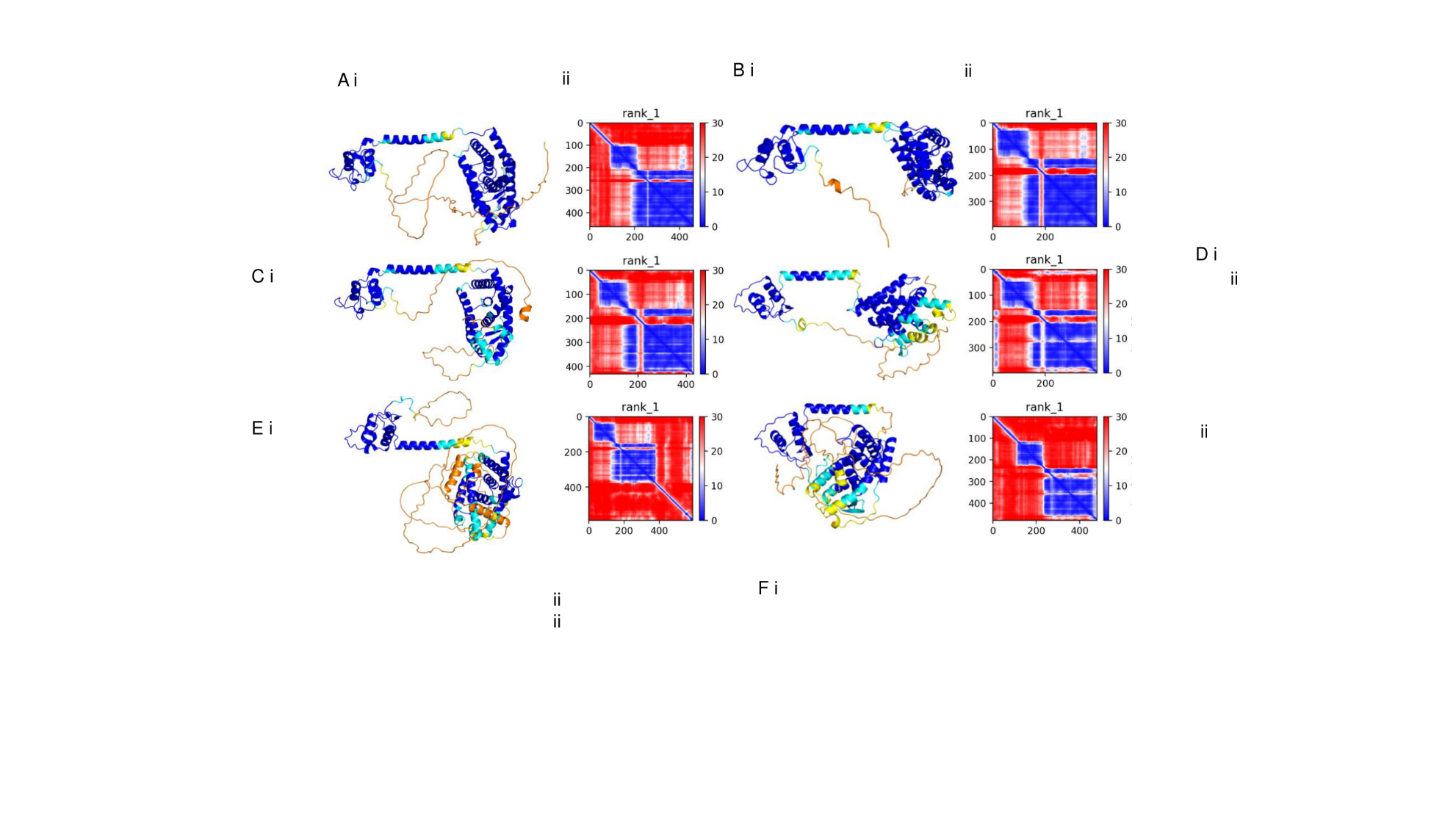
**

Figure S4.2. Structural representations of monomeric nuclear thyroid hormone receptor predictions from various species resulting from Alphafold2 modeling support high quality of generated models, particularly within functional domains. Each panel includes (i) the predicted 3D structure colored by pLDDT scores oriented with the DNA binding domain on the left and (ii) the corresponding predicted aligned error plot. Blue areas of the predicted aligned error plots represent regions where the model is confident in the relative positioning of residues within the protein structure and red areas represent lower confidence in model prediction. (A) *Homo sapiens* thyroid hormone receptor β (P10828). (B) *Danio rerio* thyroid hormone receptor β (Q9PVE4). (C) *Branchiostoma floridae* thyroid hormone receptor (A7L5U9). (D) Strongylocentrotus *purpuratus* thyroid hormone receptor β (A0A7M7N2J7). (E) *Ciona intestinalis* nuclear receptor (H2XS16). (F) *Crassostrea gigas* thyroid hormone receptor (A0A0F6V015).


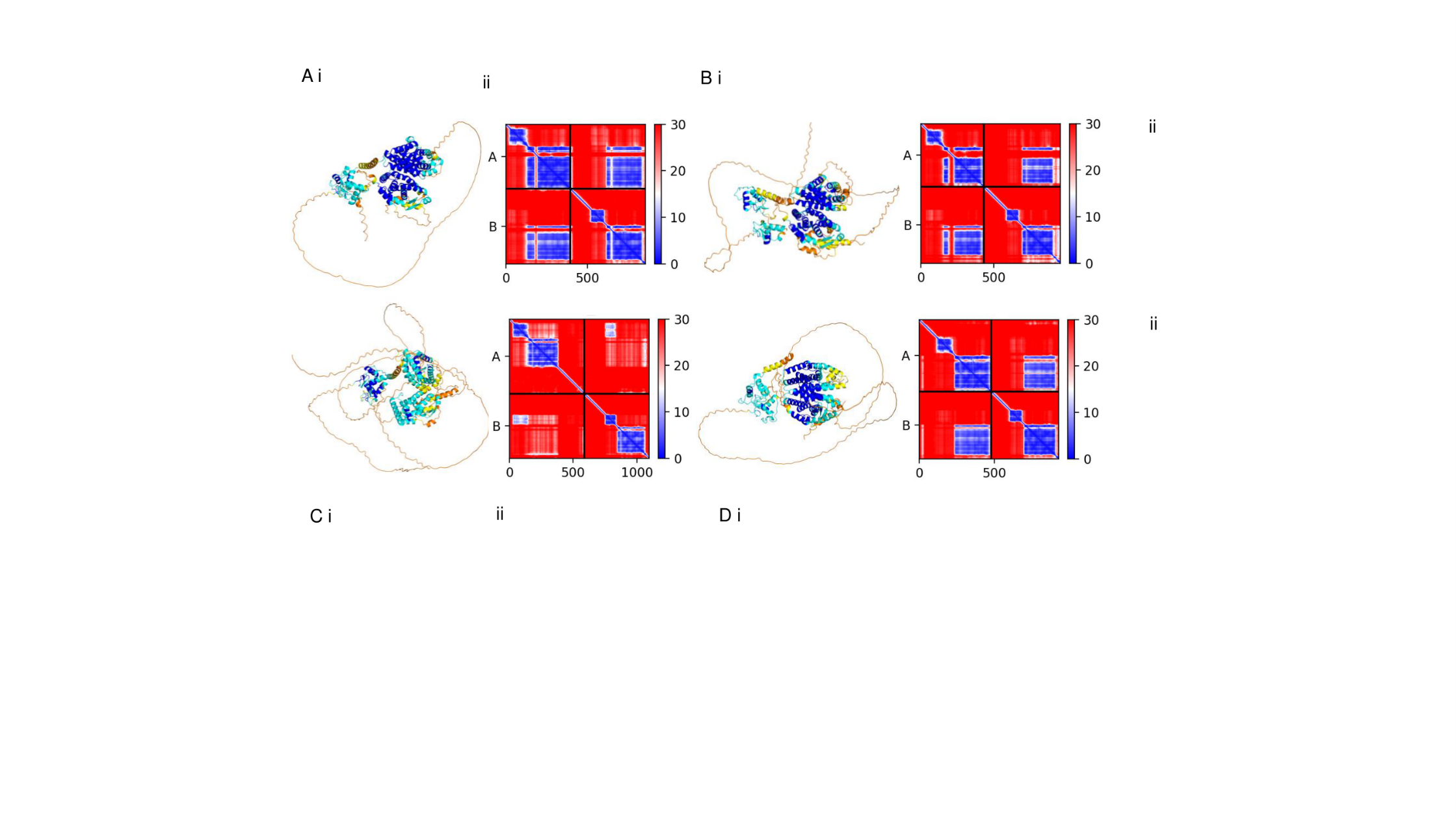


**Figure S4.3. Structural representations of dimeric nuclear thyroid hormone receptor in complex with RXR from various species resulting from Alphafold2 modeling support high quality of generated models, particularly within functional domains.** Each panel includes **(i)** the predicted 3D structure colored by pLDDT scores oriented with the DNA binding domain on the left and thyroid hormone receptor on the top of the ligand binding domain (right) and **(ii)** the corresponding predicted aligned error plot. Blue areas of the predicted aligned error plots represent regions where the model is confident in the relative positioning of residues within the protein structure and red areas represent lower confidence in model prediction. (A) *Danio rerio* thyroid hormone receptor β (Q9PVE4) + RXRαα (F1Q4V9). (B) *Branchiostoma floridae* thyroid hormone receptor (A7L5U9) + RXR (Q8MX78). (C) *Ciona intestinalis* nuclear receptor (H2XS16) + RXR (Q4H2U9). (D) *Crassostrea gigas* thyroid hormone receptor (A0A0F6V015) + RXR (K1PXX3).


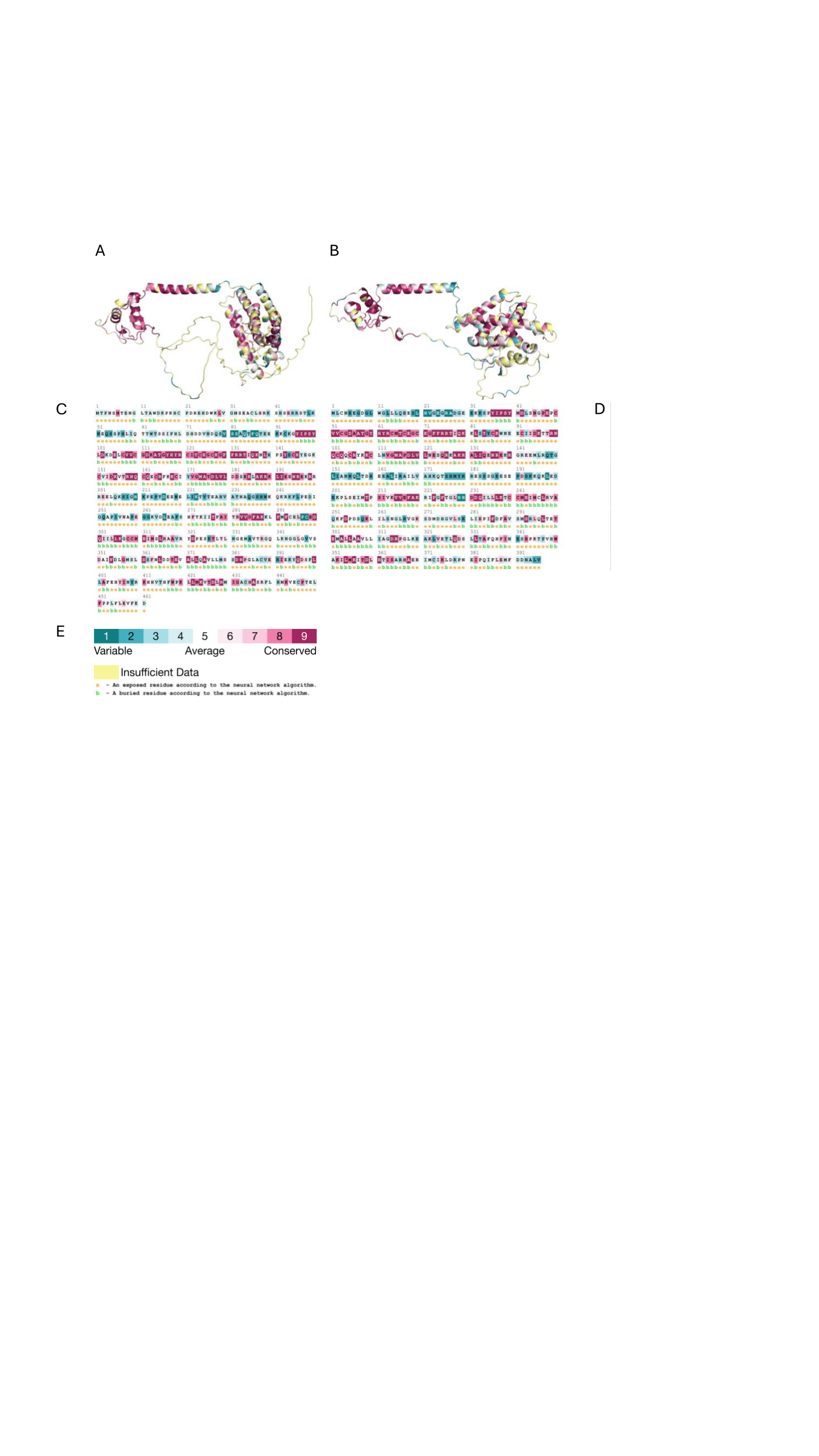


Figure S4.4. Residues are highly conserved in the DNA binding domain, along the hinge region, and in parts of the ligand binding domain between a diversity of metazoan thyroid hormone receptors. Sequences used included *Homo sapiens* thyroid hormone receptor β (P10828), *Danio rerio* thyroid hormone receptor β (Q9PVE4), *Branchiostoma floridae* thyroid hormone receptor (A7L5U9), Strongylocentrotus *purpuratus* thyroid hormone receptor β (A0A7M7N2J7), *Ciona intestinalis* nuclear receptor (H2XS16), *Crassostrea gigas* thyroid hormone receptor (A0A0F6V015). Sequences were aligned with ClustalW and figures were created with Consurf using this custom MSA. Panels (A) and (C) map conservation onto *H. sapiens* receptor while panels (B) and (D) map conservation onto *S. purpuratus* receptor for Alphafold2 predicted structure and protein sequence respectively.


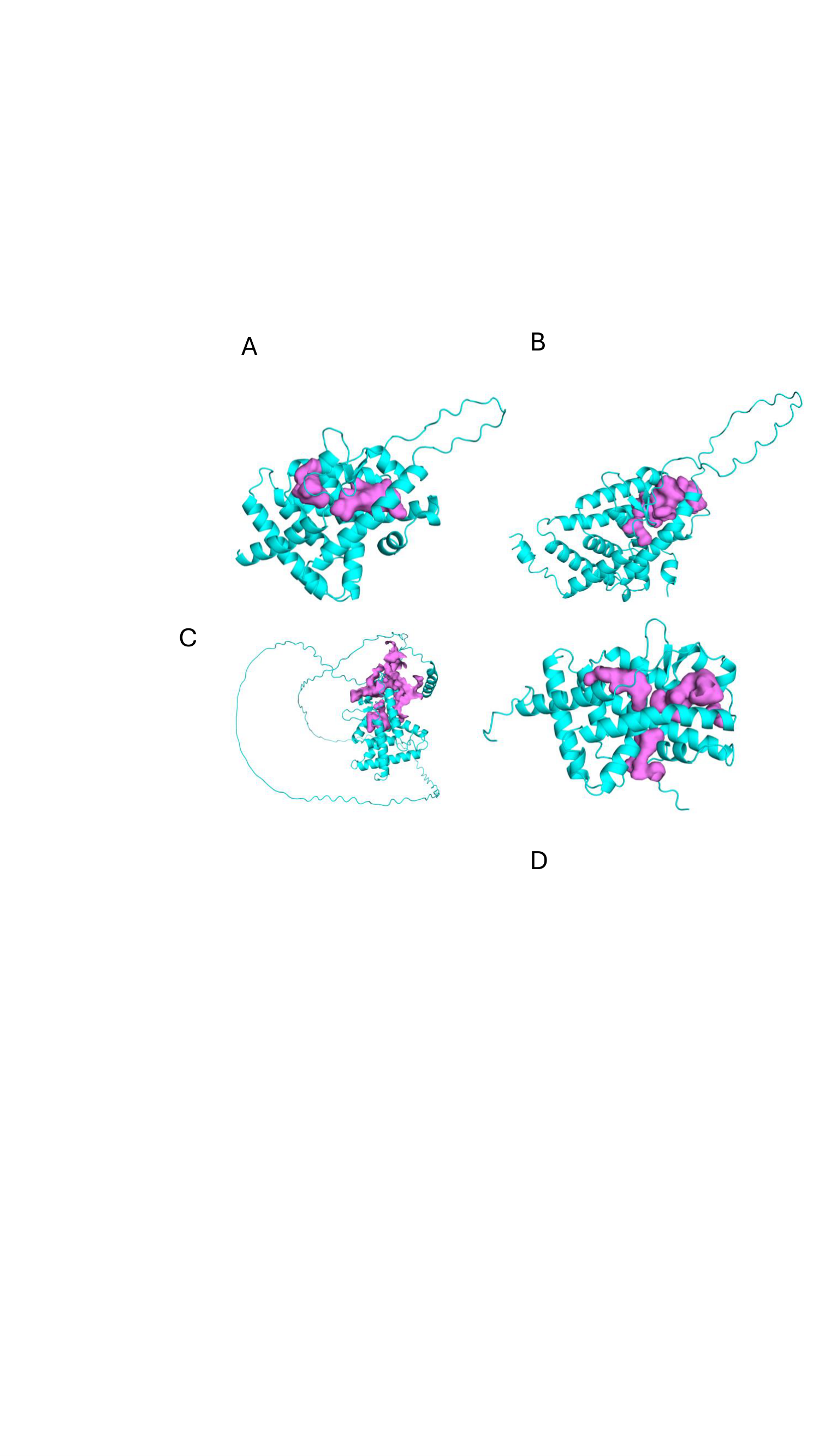


Figure S4.5. Alphafold2 predicted structures suggest that the thyroid hormone binding pocket is present, but not highly conserved across metazoans and possibly open to the outside of the protein in some invertebrates. Each panel shows the ligand binding domain of the thyroid hormone receptor from the predicted dimeric structure in cyan and large pockets and cavities within the protein in violet. (A) *Danio rerio* thyroid hormone receptor β (Q9PVE4) + RXRαα (F1Q4V9). The thyroid hormone binding pocket is shown on the right, with a second smaller pocket also shown. (B) *Branchiostoma floridae* thyroid hormone receptor (A7L5U9) + RXR (Q8MX78). There is one large cavity in this receptor which is open to the exterior of the protein. (C) *Ciona intestinalis* nuclear receptor (H2XS16) + RXR (Q4H2U9). Large cavities are found in many areas which are open to the exterior of the protein, caused by large regions protein predicted as unstructured. (D) *Crassostrea gigas* thyroid hormone receptor (A0A0F6V015) + RXR (K1PXX3). There are three large cavities in this receptor which are open to the exterior of the protein.


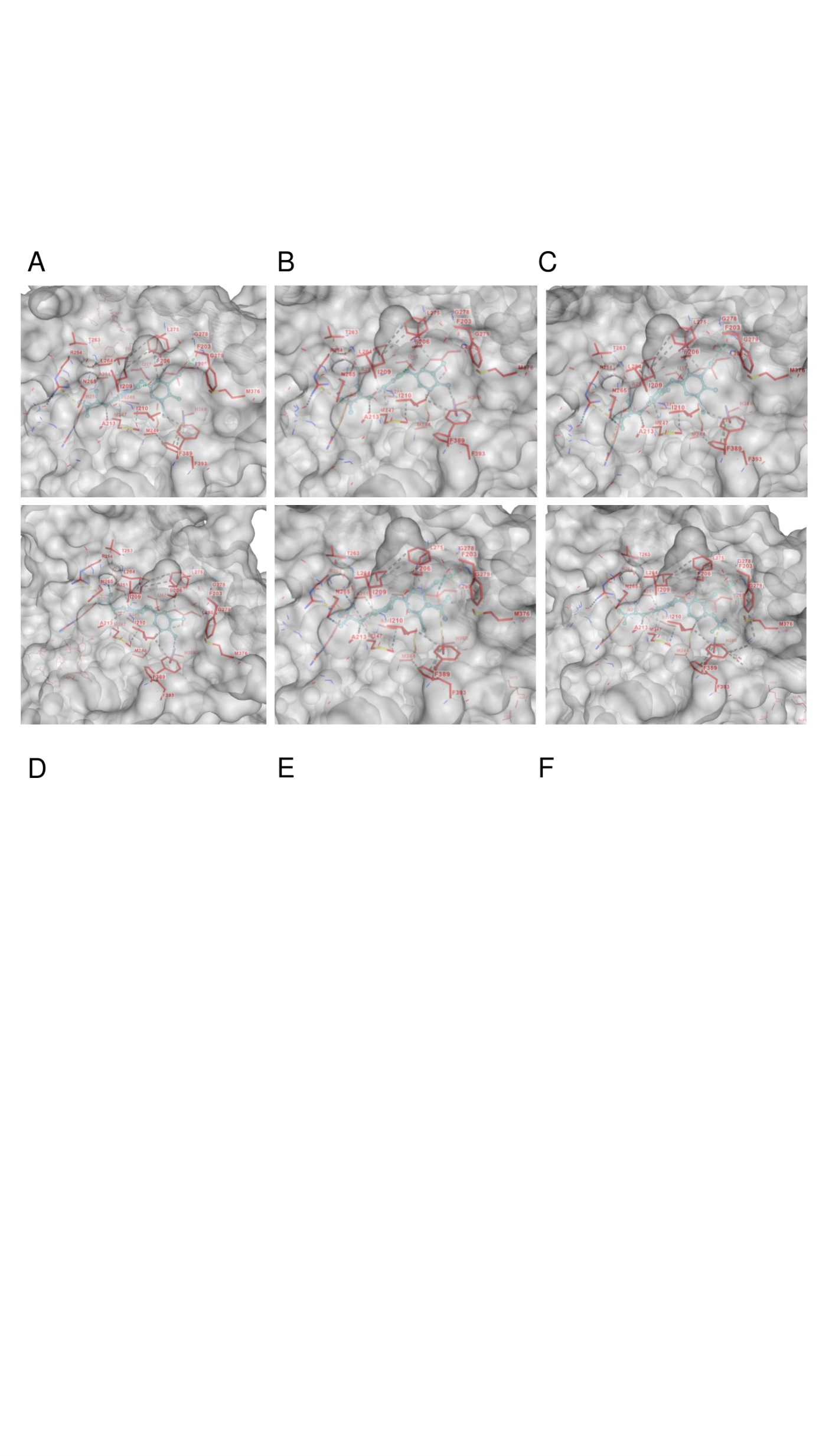


Figure S4.6. T3 binds with a higher predicted affinity than T4, rT3, TRIAC, ST4, and ST3 to the *Homo sapiens* thyroid hormone receptor β in complex with RXRα. Best binding pose for each metabolite (cyan) predicted using CBDock2 is shown with residue contacts in red. Dotted lines depict predicted bonds between residues and the ligand. (A) T4 bound with a predicted binding affinity of -7.1. (B) T3 bound with a predicted binding affinity of -9.5. (C) rT3 bound with a predicted binding affinity of -6.8. (D) TRIAC bound with a predicted binding affinity of -9. (E) ST4 bound with a predicted binding affinity of -5.3. (F) ST3 bound with a predicted binding affinity of -7.9.


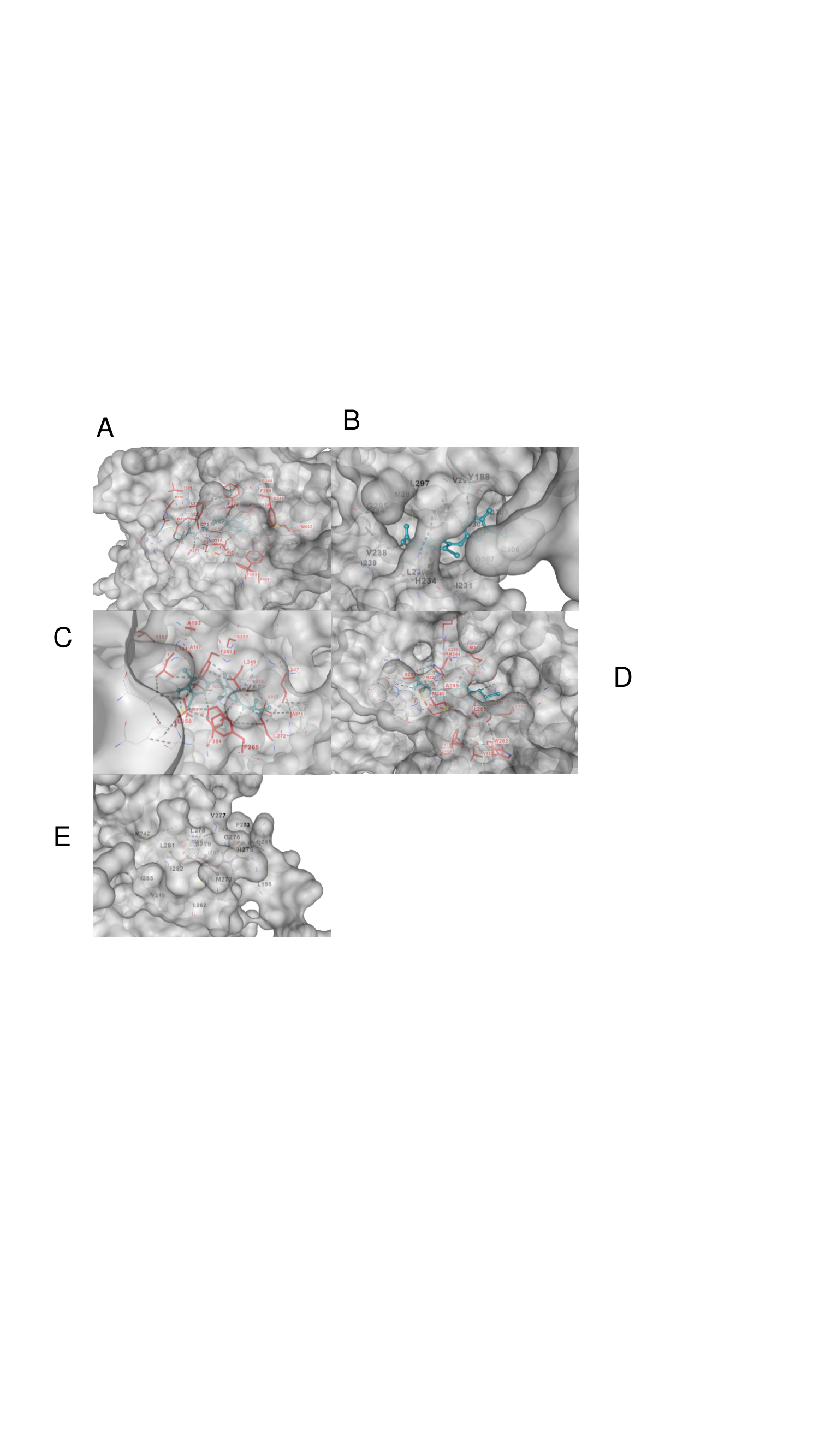


Figure S4.7. Binding poses of TH metabolites with THRs from various species. Best binding pose for each metabolite (cyan) predicted using CBDock2 is shown with residue contacts in red or grey. Dotted lines depict predicted bonds between residues and the ligands. (A) Danio rerio dimer with T4 bound in a buried binding pocket. (B) *Branchiostoma floridae* dimer with TRIAC bound in an open binding cavity. (C) *Crassostrea gigas* dimer with T4 bound in an open binding cavity. (D) *Ciona intestinalis* dimer with T4 bound in an open binding cavity. (E) *Strongylocentrotus purpuratus* dimer with T4 bound in an open binding cavity.

Table S4.1. Scores of top nuclear thyroid hormone receptor monomer and dimer models for six species generated from amino acid sequence by Alphafold2. pLDDT stands for predicted local distance difference test and is the calculated confidence for each residue averaged across the protein. pTM stands for predicted template modeling score and represents overall model confidence. ipTM stands for interface pTM and represents confidence of protein-protein interaction.

| Receptor | Species | pLDDT | pTM | ipTM |
| --- | --- | --- | --- | --- |
| THRβ | *Homo sapiens* | 79.5 | 0.612 | N/A |
| THRβ | *Danio rerio* | 87.9 | 0.682 | N/A |
| THR | *Branchiostoma floridae* | 81.2 | 0.624 | N/A |
| THRβ | *Strongylocentrotus purpuratus* | 80.9 | 0.623 | N/A |
| NR | *Ciona intestinalis* | 64.3 | 0.498 | N/A |
| THR | *Crassostrea gigas* | 71.6 | 0.51 | N/A |
| THRβ+RXRα | *Homo sapiens* | 65.8 | 0.541 | 0.565 |
| THRβ+RXRαα | *Danio rerio* | 69.4 | 0.578 | 0.637 |
| THR+RXR | *Branchiostoma floridae* | 60.7 | 0.514 | 0.557 |
| THRβ+RXR | *Strongylocentrotus purpuratus* | 73.4 | 0.604 | 0.618 |
| NR+RXR | *Ciona intestinalis* | 53 | 0.354 | 0.34 |
| THR+RXR | *Crassostrea gigas* | 62.1 | 0.517 | 0.512 |

Table S4.2. Predicted CBDock2 binding affinity of thyroid hormone metabolites to thyroid hormone receptor reveals differences in binding between monomeric and dimeric models, and differences in binding affinity between species and metabolites.

| Receptor | ΔG T4 | ΔG T3 | ΔG rT3 | ΔG TRIAC | ΔG ST4 | ΔG ST3 |
| --- | --- | --- | --- | --- | --- | --- |
| *Homo sapiens* monomer | -6.5 | -8.1 | -7.8 | -7.9 | -3.6 | -6.4 |
| *Homo sapiens* dimer | -7.1 | -9.5 | -6.8 | -9 | -5.3 | -7.9 |
| *Danio rerio* monomer | -7.3 | -9.4 | -7.6 | -8.9 | -5.4 | -9.4 |
| *Danio rerio* dimer | -5.4 | -9 | -7.8 | -8.2 | -3.8 | -7.1 |
| *Branchiostoma floridae* monomer | 0.4 | -1.5 | -2.2 | -3.7 | 1.6 | -1.2 |
| *Branchiostoma floridae* dimer | -6.3 | -7.6 | -6.2 | -7.7 | -5.7 | -6.2 |
| *Strongylocentrotus purpuratus* monomer | 76.2 | 63 | 54.5 | 56.7 | 71.5 | 54.4 |
| *Ciona intestinalis* monomer | 85.5 | 83.9 | 71.3 | 109.9 | 68.8 | 68.3 |
| *Ciona intestinalis* dimer | 78.8 | 58.3 | 62.6 | 66.8 | 79.5 | 71.3 |
| *Crassostrea gigas* monomer | 1.4 | -1.7 | 0.8 | 0.5 | 2 | -1 |
| *Crassostrea gigas* dimer | 33.4 | 39.7 | 33.4 | 32.5 | 59 | 53.9 |
